# PdeH, a High-Affinity cAMP Phosphodiesterase, Is a Key Regulator of Asexual and Pathogenic Differentiation in *Magnaporthe oryzae*

**DOI:** 10.1371/journal.ppat.1000897

**Published:** 2010-05-06

**Authors:** Ravikrishna Ramanujam, Naweed I. Naqvi

**Affiliations:** 1 Fungal Patho-Biology Group, Temasek Life Sciences Laboratory, Singapore; 2 School of Biological Sciences, Nanyang Technological University, Singapore; 3 Department of Biological Sciences, National University of Singapore, Singapore; University of Melbourne, Australia

## Abstract

Cyclic AMP-dependent pathways mediate the communication between external stimuli and the intracellular signaling machinery, thereby influencing important aspects of cellular growth, morphogenesis and differentiation. Crucial to proper function and robustness of these signaling cascades is the strict regulation and maintenance of intracellular levels of cAMP through a fine balance between biosynthesis (by adenylate cyclases) and hydrolysis (by cAMP phosphodiesterases). We functionally characterized gene-deletion mutants of a high-affinity (PdeH) and a low-affinity (PdeL) cAMP phosphodiesterase in order to gain insights into the spatial and temporal regulation of cAMP signaling in the rice-blast fungus *Magnaporthe oryzae*. In contrast to the expendable PdeL function, the PdeH activity was found to be a key regulator of asexual and pathogenic development in *M. oryzae*. Loss of PdeH led to increased accumulation of intracellular cAMP during vegetative and infectious growth. Furthermore, the *pdeH*Δ showed enhanced conidiation (2–3 fold), precocious appressorial development, loss of surface dependency during pathogenesis, and highly reduced *in planta* growth and host colonization. A *pdeH*Δ *pdeL*Δ mutant showed reduced conidiation, exhibited dramatically increased (∼10 fold) cAMP levels relative to the wild type, and was completely defective in virulence. Exogenous addition of 8-Br-cAMP to the wild type simulated the *pdeH*Δ defects in conidiation as well as *in planta* growth and development. While a fully functional GFP-PdeH was cytosolic but associated dynamically with the plasma membrane and vesicular compartments, the GFP-PdeL localized predominantly to the nucleus. Based on data from cAMP measurements and Real-Time RTPCR, we uncover a PdeH-dependent biphasic regulation of cAMP levels during early and late stages of appressorial development in *M. oryzae*. We propose that PdeH-mediated sustenance and dynamic regulation of cAMP signaling during *M. oryzae* development is crucial for successful establishment and spread of the blast disease in rice.

## Author Summary

*Magnaporthe oryzae*, an economically important fungal pathogen that causes the blast disease in rice and several cereal crops, is a model organism for studying fungus-host interactions. *M. oryzae* reproduces asexually by producing spores, which can switch to an infectious disease-causing mode of development in response to host and environmental cues. Subsequent activation of conserved signaling modules involving the second messenger cyclic AMP has been shown to be important for pathogenic development. In this study, we used a variety of genetic and biochemical approaches to functionally characterize two enzymes (encoded by *PDEH* and *PDEL* genes in *M. oryzae*) which regulate the intracellular levels of cAMP signaling through hydrolysis. Furthermore, we show that the timely modulation and maintenance of appropriate levels of cAMP is essential for proper asexual development, and more importantly for the ability of *M. oryzae* to successfully infect and cause disease. Our results suggest that cyclic AMP signaling is broadly compartmentalized (nuclear and cytosolic) and that *M. oryzae* is fully capable of inducing and responding rapidly to short pulses of cAMP in the various cell types related to the above developmental events.

## Introduction

Heterotrimeric G protein signaling utilizes cyclic AMP (cAMP) as a second messenger, to mediate the transduction of extracellular stimuli to the intracellular downstream signaling components in several eukaryotes, including the pathogenic fungi. The cAMP pathway is a highly conserved signaling module that influences and regulates a range of fundamental cellular processes in growth, development and morphogenesis.

In response to ligand-stimulated GPCRs, spatially segregated “point sources” of cAMP are generated through Gα_S_ based activation of membrane anchored adenylyl cyclases. In response to extracellular stimuli, multiple point sources of cAMP are generated throughout the cell, leading to the gradual accumulation and increase in the basal or steady-state levels of cAMP. On attaining a critical threshold concentration, cAMP can further activate several important effectors (foremost being cPKA, a cAMP-dependent Protein Kinase A), which in turn mediate a wide array of downstream physiological effects [1]. The inactivation of cAMP to 5′-AMP is carried out through enzymatic hydrolysis, by phosphodiesterases (PDEs). Such an inactivation of cAMP regulates the overall strength and intensity of the signaling cascade and is also necessary for efficient signal compartmentalization and termination [2], [3]. In order to achieve this, PDEs are targeted to specific intracellular sites or signaling complexes and are known to localize not only to the cytosol, but also to a variety of membrane, nuclear and cytoskeletal locations [4], [5], [6], [7], [8]. In addition, PDEs establish and shape concentration-dependent gradients of cAMP at distinct regions within a cell [9], [10], [11]. Thus, PDEs play an important role in regulating the specificity, amplitude and temporal duration of cAMP signaling [2], [12].

In fungi and yeasts, cAMP signaling cascade has been co-opted for a multitude of cellular processes and development. For example, in yeasts like *S. cerevisiae*, cAMP regulates nutrient sensing, pseudohyphal differentiation, cell cycle progression and stress signaling [13], [14], [15], [16], [17], [18]. In *S. pombe*, mating, sporulation and gluconeogenesis are controlled through the cAMP pathway [19], [20], [21], [22]. In pathogenic fungi, such as *C. albicans* and *C. neoformans*, cAMP signaling influences sexual differentiation, stress tolerance and several important aspects of virulence [23], [24], [25], [26], [27], [28], [29], [30], [31], [32], [33]. In the plant-pathogenic fungus *U. maydis*, cAMP governs dimorphic transition in addition to virulence [34], [35], [36], [37], [38]. Morphogenesis, cell polarity and asexual development are regulated through cAMP in *N. crassa and A. nidulans* [39], [40], [41], [42], [43], [44], [45], [46], [47], [48], [49].

Fluctuations in cAMP levels are modulated by cAMP phosphodiesterases in yeasts (including the dimorphic pathogens) but not in filamentous fungal species. *S. cerevisiae* contains a low-affinity phosphodiesterase (Pde1; with a high K_m_ towards cAMP) and a high-affinity phosphodiesterase (Pde2; with a low K_m_ towards cAMP) [50], [51], [52], [53]. Pde1 regulates cAMP levels induced by glucose stimulation or intracellular acidification, and is in turn regulated through phosphorylation by cPKA [54], [55], [56].

Pde2 regulates basal or steady state levels of cAMP, in addition to protecting the yeast cells from extracellular cAMP [17], [54], [57]. Although poorly understood, Pde2 regulation is mediated through the cPKA pathway. Neither of the two PDEs is indispensable for cell growth under standard culture conditions but are primarily needed to overcome stress and nutritional starvation [17], [58], [59]. The fission yeast *S. pombe*, lacks a Pde2 ortholog, but possesses a Pde1 protein, which functions to regulate cAMP levels induced by glucose stimulation in a manner likely dependent on cPKA [21], [60]. A definite role has not yet been assigned to the Pde1 in *C. albicans* development. However, the *C. albicans* Pde2 has been functionally well characterized [23], [25], [61]. Similar to the effects caused in *S. cerevisiae*, deletion of Pde2 leads to elevated levels of intracellular cAMP, increased responsiveness to exogenous cAMP and sensitivity to heat shock [23], [27]. In addition, Pde2 mutant displays defects in cell wall and membrane integrity, and is more sensitive to a range of antifungal agents [24], [25]. Furthermore, the pde2 mutant shows enhanced filamentation but is avirulent in a murine model of systemic Candidiasis [23].

The Pde1 and Pde2 have been functionally characterized in *Cryptococcus neoformans*, which represents another well-studied human fungal pathogen. Unlike in *S. cerevisiae* and *C. albicans*, Pde2 deletion in *C. neoformans* results in only subtle and mild phenotypic defects. In contrast, Pde1 regulates the basal levels of cAMP but does not respond to cAMP induced in response to glucose induction. However, deletion of *PDE1* conferred only mild phenotypic defects. Furthermore, Pde1 activity is regulated through cPKA derived phosphorylation [30].

Mouse strains deficient in PDE activity are viable, but exhibit poor growth, increased prenatal mortality or female infertility [62], [63]. Taken together, the phenotypes displayed by the PDE deletion strains in either fungi or in mammals, imply that, rather than executing a strict regulatory role, PDEs function to modulate and streamline cAMP signaling.

*M. oryzae* is an ascomycete fungus that causes the blast disease in rice and several other monocot species [64]. It reproduces asexually when stimulated by light, typically producing three to four conidia each on stalk-like structures known as conidiophores [65], [66], [67]. These asexual spores or conidia aid in the spread of the blast disease. Under conditions of high humidity, the asexual spore produces a germ tube which senses and responds to host surface stimuli by forming an infection structure known as an appressorium [68]. In *M. oryzae*, cAMP signaling is required for conidiation and appressorium initiation [69], [70], [71]. Although an Adenylate cyclase (mac1) function has been shown to be crucial for *M. oryzae* pathogenesis, key downstream modulators of cAMP signaling are yet to be identified [72]. In this study, we were interested in deciphering the roles of phosphodiesterases in modulating the cAMP levels and signaling during various stages of *M. oryzae* development. Towards this end, we identified a high- and low-affinity cAMP phosphodiesterase in *M. oryzae* and further characterized gene-deletion strains of both the cAMP phosphodiesterases. Our results show that PdeH plays an important role in regulating the steady-state levels of cAMP during asexual, pathogenic and invasive growth in *M. oryzae*. We analyzed the transcriptional regulation and localization of PDEs and demonstrate that cAMP signaling is compartmentalized in *M. oryzae* and that it responds rapidly to modulate and maintain the appropriate levels of cAMP during growth, development and infection.

## Results

### Identification of cAMP phosphodiesterases in *M. oryzae*

**Figure 1:**
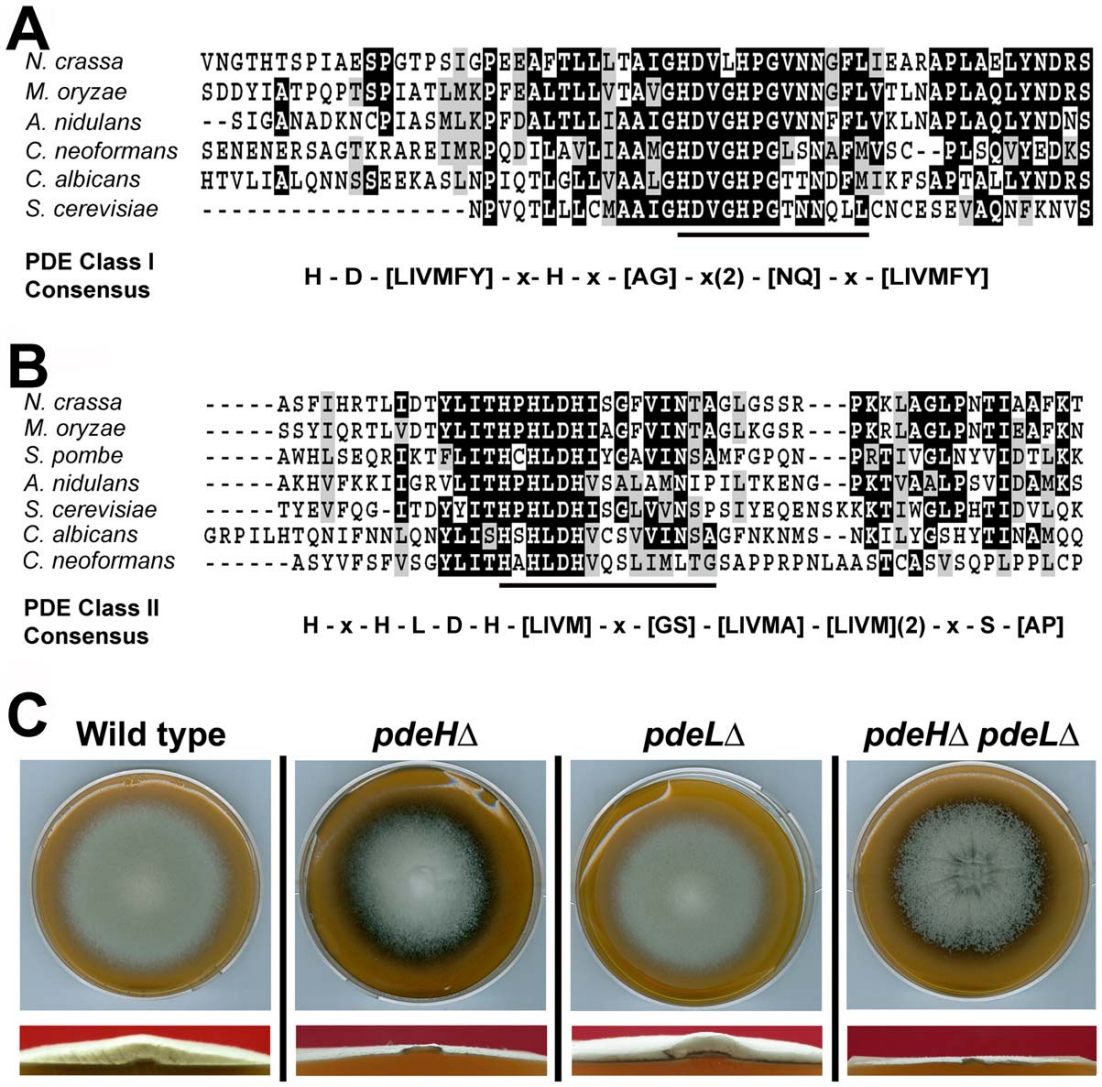
Identification and deletion analysis of cAMP phosphodiesterase genes in *M. oryzae*. (**A**) Sequence alignment of the predicted active sites of the high-affinity cAMP phosphodiesterase from *N. crassa* (NCU00478), *M. oryzae* (MGG_05664), *A. niger* (AN2740.2), *S. cerevisiae* (CAA99689), *C. albicans* (AAM89252) *and C. neoformans* (AY874131). The conserved PDE Class I consensus is represented below. (**B**) Comparative alignment of the region containing the predicted signature sequence of the low-affinity cAMP phosphodiesterase from *N. crassa* (NCU00237), *M. oryzae* (MGG_07707), *S. pombe* (CAA20842), *A. niger* (AN0829.2), *S. cerevisiae* (CAA64139), *C. albicans* (XP720545) and *C. neoformans* (AY864841). The Class II PDE consensus sequence is underlined and depicted below in detail. *S. pombe* lacks the high affinity PDE variant. The multiple sequence alignment was performed using Clustal W and Boxshade (http://bioweb.pasteur.fr/seqanal/interfaces/boxshade.html). The conserved amino acid residues are shaded black, whereas similar residues are shown in gray. (**C**) PdeH is necessary for proper aerial hyphal growth. Morphology of the wild-type, *pdeH*Δ, *pdeL*Δ or the *pdeH*Δ *pdeL*Δ colonies. The indicated strains were grown in the dark on prune agar medium for a week and photographed (Upper panel). The lower panels show the cross sections of the above colonies at near-median planes. The *pdeH*Δ and the *pdeH*Δ *pdeL*Δ are dramatically reduced in aerial hyphal growth.

Using complete amino acid sequences, we identified orthologs of the yeast Pde1 and Pde2 in *M. oryzae* (http://www.broadinstitute.org/annotation/genome/magnaporthe_grisea/MultiHome.html). MGG_05664 ORF (GQ869475) was predicted to encode an 893 aa protein, showing 27% identity to Pde2; whereas MGG_07707 (GQ869476) was a 585 aa polypeptide with 32% overall identity to yeast Pde1. The complete cDNA sequence was determined in each instance (Genbank accession GQ869476 and GQ869476, respectively) and the predicted amino acid sequences confirmed. MGG_05664 showed the conserved PDE class I consensus sequence [73] permitting us to designate it as a high-affinity phosphodiesterase (Figure 1A). Based on the conserved PDE class II consensus [30], MGG_07707 was likewise judged to be the low-affinity cAMP phosphodiesterase in *M. oryzae* (Figure 1B). In order to avoid confusion with Pde1, a P-Type ATPase [74], we hereafter refer to the low-affinity phosphodiesterase as PdeL and the high-affinity phosphodiesterase as PdeH in *M. oryzae*.

### *PDEH* is necessary for proper aerial hyphal growth

To understand the role of cAMP phosphodiesterases during growth and morphogenesis in *M. oryzae*, we generated gene-deletion mutants of *PDEH* and *PDEL* in the B157 wild type background. *Agrobacterium*-mediated gene targeting was used to replace the entire ORF of the *PDEH* or *PDEL* gene with the hygromycin-resistance (Figure S1A) or the bialaphos-resistance marker cassettes (Figure S1B), respectively. Selected transformants were screened by locus-specific PCR and confirmed by Southern blotting (Figure S1C and S1D). Two independent strains for *pdeH*Δ and *pdeL*Δ were used for further investigations.

When grown on Prune agar (PA) medium for 7 days at 28°C, the *pdeL*Δ was similar to the wild-type strain and showed no obvious defects in aerial or radial growth and colony morphology (Figure 1C). In contrast, the *pdeH*Δ colony was flat due to reduced aerial hyphal growth, and displayed enhanced pigmentation and marginally slower radial growth at analogous time points (Figure 1C). To further understand the relationship between *PDEH* and *PDEL*, we generated a *pdeH*Δ *pdeL*Δ double mutant (Figure S1E). Compared to the *pdeH*Δ, the double deletion mutant showed a flat colony appearance with enhanced pigmentation and severely reduced aerial hyphal growth (Figure 1C and S3A), thus underscoring the importance of proper PdeH and PdeL function in *M. oryzae*. In addition, radial growth was compromised in the double mutant. Next, we attempted a genetic complementation of *pdeH*Δ by introducing a full-length *PDEH* fused to RFP at the N-terminus under native regulation. The complemented *pdeH*Δ strain showed complete suppression of the aerial and radial growth defects exhibited by the *pdeH*Δ strain (Figure S2A and 2B), suggesting that the phenotypic defects therein were solely due to the loss of PdeH function. Taken together, these results indicate that PdeH is essential for proper aerial hyphal growth and development in *M. oryzae*. Furthermore, we infer that PdeL plays only a minor role in regulating vegetative and aerial growth in *M. oryzae*.

### Loss of PdeH Leads to enhanced conidiation

**Figure 2:**
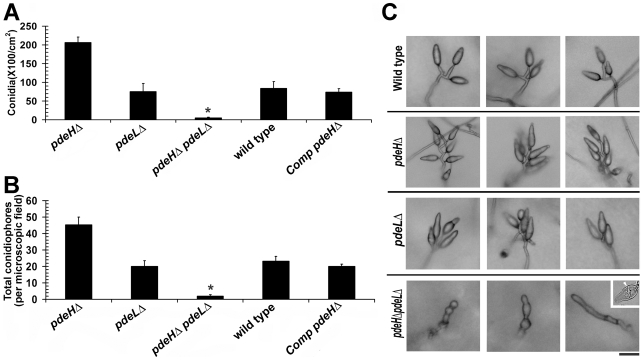
PdeH function regulates asexual development in *M. oryzae*. Comparative quantitative analysis of conidiation (**A**) and conidiophores (**B**) in the wild type, *pdeH*Δ, *pdeL*Δ, *pdeH*Δ *pdeL*Δ and the complemented *pdeH*Δ strain (Comp *pdeH*Δ; RFP-PdeH expressing *pdeH*Δ). (**A**) For the assessment of conidiation, the respective strains were initially grown in the dark for a day followed by exposure to constant illumination for 6 d. Data represents mean ± SE based on five independent replicates. (**B**) The number of conidiophores produced by the indicated strains per microscopic field was quantified 24 h post photo induction. Results were quantified in three independent replicates and represented as mean ± SE. Asterisk highlights the heavily pigmented aberrant structures of varied morphologies produced by the double deletion mutant during conidiation phase. The quantification represents conidia-like or aberrant conidiophore like structures present therein. (**C**) Total number and arrangement of conidia on conidiophores in the wild type, *pdeH*Δ, *pdeL*Δ and the *pdeH*Δ *pdeL*Δ. The indicated strains were grown on PA medium layered on a glass slide. The strains were initially incubated in the dark for a day and then subjected to constant illumination for a period of 3 d prior to microscopic observations. Inset depicts the rare two celled conidia-like structure formed by the double deletion mutant. The arrow points to the single septum in the aberrant structure. Scale bar = 25 micron.

Asexual reproduction commences in *M. oryzae* with the formation of specialized aerial conidiophores that go on to produce conidia in a sympodial manner [65]. We were particularly curious about the conidiation status of *pdeH*Δ since it showed reduced aerial growth. Rather surprisingly, conidiation was two to three-fold higher in the *pdeH*Δ, when compared to *pdeL*Δ or the wild type (Figure 2A). Furthermore, the *pdeH*Δ formed 5–8 conidia per conidiophore unlike the wild type conidiophores, which typically produce 3 conidia each (Figure 2C). The *pdeL*Δ *pdeH*Δ strain interestingly was severely blocked in conidiation (98±1.0% reduced in conidiation when compared to the wild type) and failed to produce proper conidia and instead formed highly pigmented aberrant structures of varied morphologies (Figure 2A, 2C and S3A) or two celled conidia-like structures at a very low frequency (≤2%; Figure 2C inset and S3C; p<0.05). Next, we quantified conidiophore formation in the above four strains and found that the *pdeH*Δ produced twice the number of conidiophores per microscopic field, compared to the *pdeL*Δ or the wild type (Figure 2B). The *pdeH*Δ *pdeL*Δ was also defective in conidiophore differentiation, and resulted in highly pigmented structures that were misshapen and failed to grow further and form conidia (Figure 2B, 2C and S3A). The *pdeL*Δ and the complemented *pdeH*Δ (RFP-PdeH expressing strain) produced nearly the same number of conidia and conidiophores as the wild type and did not show any apparent defects during any stage of asexual development (Figure 2A and 2B).

Taken together, our results suggest that PdeH regulates multiple aspects of asexual development, primarily influencing conidiophore differentiation and conidia formation. Furthermore, we concur that PdeH function (and by inference cAMP levels) likely determines the pattern and number of conidia formed on conidiophores. Furthermore, we conclude that the simultaneous loss of PDE functions leads to deleterious effects in asexual differentiation in *M. oryzae*. The inability of the *pdeH*Δ *pdeL*Δ to form proper conidiophores and conidia suggests a regulatory albeit ancillary function for PdeL during asexual development.

### PdeH regulates intracellular cAMP levels in *M. oryzae*

**Figure 3:**
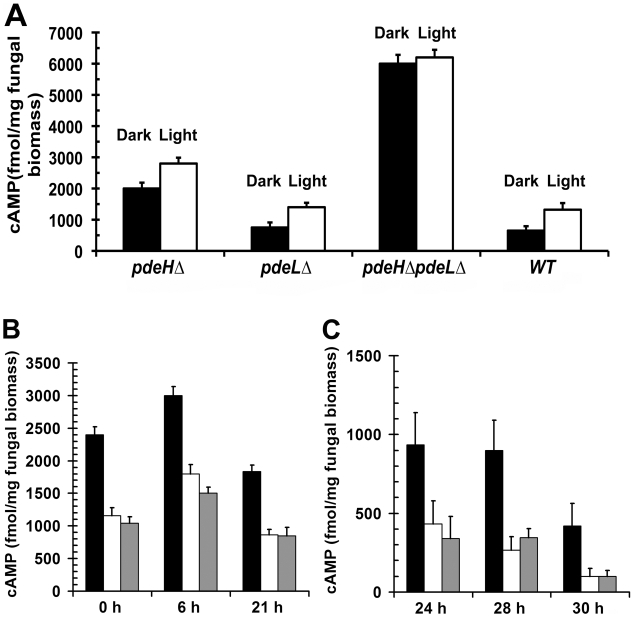
PdeH regulates the intracellular cAMP levels during growth and development in *M. oryzae*. Bar graphs depicting the steady-state levels of cAMP prevalent in the *pde*HΔ, *pdeL*Δ, *pdeH*Δ *pdeL*Δ and wild type during asexual development and pathogenesis. (**A**) Estimation of the cAMP levels in the indicated strains during asexual development (aerial hyphae, conidiophores and conidia). The indicated strains were initially incubated in the dark (black bar; non-conidiating) for 3 d, followed by exposure to light for 24 h (white bar; conidiating) prior to cAMP measurements. Values represent mean ± S.E from two independent experiments with two replicates each. (**B**) Conidia from the *pdeH*Δ (black bar), *pdeL*Δ (white bar) and wild type (gray bar) strains were inoculated on inductive surface of GelBond membranes and harvested at the indicated time points for cAMP estimations. The 0 h time point represents freshly harvested conidia, while 6 h to 21 h represents the early stage of pathogenic development. Values represent mean ± S.E from three independent experiments with three replicates each. (**C**) The steady-state levels of cAMP were also quantified from conidia inoculated on inductive surfaces for 24, 28, or 30 h representing the late stages of pathogenic development. Values represent mean ± S.E from three separate experiments with three replicates each.

In order to assess whether the phenotypic defects in *pdeH*Δ were due to elevated levels of cAMP, we quantified and compared the steady-state levels of cAMP during the conidiation phase in the *pdeH*Δ, *pdeL*Δ, *pdeH*Δ *pdeL*Δ and the wild type (Figure 3A). First, we measured cAMP levels in cultures grown in the dark for 3 d and compared it to those in cultures exposed to constant illumination for a period 24 h (after a 3 d incubation in the dark). We found that even in the absence of light stimulation (darkness), the *pdeH*Δ strain accumulated ∼3 fold (p<0.005) and ∼2.6 fold (p<0.005) higher levels of cAMP, compared to the wild type or the *pdeL*Δ respectively (Figure 3A). Under similar conditions, the double deletion mutant accumulated ∼3 fold (p<0.001) higher cAMP compared to *pdeH*Δ and ∼10 fold higher levels compared to the wild type or *pdeL*Δ (p<0.001).

Furthermore, during conidiation (24 h photo-induced) the cAMP levels were significantly up regulated in all the strains except the double deletion mutant: 2 fold (p<0.05) in the wild type and *pdeL*Δ, and by 1.4 fold (p<0.01) in the *pdeH*Δ when compared to the respective dark grown cultures. However, in the *pdeH*Δ *pdeL*Δ, the cAMP levels were consistently higher, irrespective of the growth conditions (Figure 3A). Thus, the *pdeH*Δ *pdeL*Δ and *pdeH*Δ mutants accumulate significantly higher levels of cAMP compared to the wild type or *pdeL*Δ. We thus conclude that PdeH is an important regulator of cAMP signaling during asexual development in the rice blast fungus.

Next, we measured the steady-state levels of cAMP in the *pdeH*Δ, *pdeL*Δ and the wild type during pathogenic development (Figure 3B and 3C). The intracellular levels of cAMP could not be estimated reliably during the pathogenic phase of development in the *pdeH*Δ *pdeL*Δ strain, as it failed to conidiate properly and the resultant aberrant structures were scarce.

The various developmental stages and time points that we considered for the cAMP measurements were as follows: ungerminated conidia (0 h), appressorium initiation (6 h), mature appressorium (21 h), penetration stage (24 h) and infection hyphae formation stage (28–30 h)[75]. The levels of cAMP were ∼2.5 fold (p<0.005) higher in freshly harvested *pdeH*Δ conidia when compared to the wild type or the *pdeL*Δ (Figure 3B). At 6 hours post inoculation (hpi), the aforementioned strains accumulated higher levels of cAMP, but the overall level was significantly higher in the *pdeH*Δ (1.5 fold; p<0.005). In mature appressoria (21 hpi) the cAMP levels decreased across all the strains, except the *pdeH*Δ, which sustained a two-fold higher level of cAMP compared to wild type or *pdeL*Δ (Figure 3B). At 24 hpi, the cAMP levels decreased further by two-fold across all the strains with the exception of the *pdeH*Δ, which continued to accumulate elevated cAMP levels. At the stage of host penetration (24–30 hpi), the *pdeH*Δ maintained comparatively high cAMP levels (2 to 5 fold; Figure 3C) unlike the wild type or *pdeL*Δ. We therefore infer that the overall steady-state levels of cAMP are regulated in two distinct phases in *M. oryzae*: upregulated during the early stages (appressorium initiation and formation), while being down regulated at the late stages (host invasion) of pathogenic development.

We thus conclude that PdeH-based regulation helps maintain functionally relevant levels of cAMP during infection-related morphogenesis. We propose a minor role for PdeL in regulating cAMP levels in *M. oryzae*. However, based on the cAMP levels observed in the *pdeH*Δ *pdeL*Δ and the *pdeH*Δ, we suggest that PdeL function likely gains more importance in the absence of PdeH.

### PdeH and surface dependency during appressorium formation

**Figure 4:**
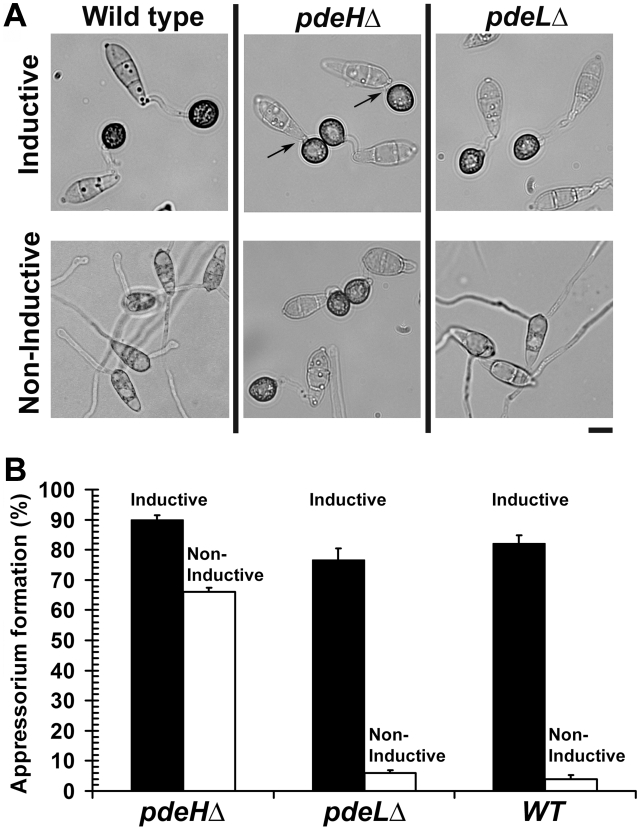
*PDEH* deletion abolishes surface dependency during pathogenesis. Appressorium formation assays on inductive and non-inductive surfaces. (**A**) Wild-type, *pdeH*Δ or *pdeL*Δ conidia were inoculated on inductive (plastic cover slips) or non-inductive surfaces (GelBond membrane) and assessed for appressoria formation after 12 h. In addition to forming appressoria on inductive surface, the *pdeH*Δ strain elaborated melanized appressoria on non-inductive surface too. The arrows highlight extremely short or negligible germ tubes formed by the *pdeH*Δ conidia on inductive plastic cover slips. Scale bar = 10 micron. (**B**) Bar graph depicting the efficiency of appressorium formation in the *pdeH*Δ, *pdeL*Δ and wild type on inductive (black bar) or non-inductive surface (white bar). Values represent mean ± S.E from three independent replicates involving 1000 conidia per sample.

Inductive surface cues such as hardness and hydrophobicity, which naturally prevail on leaves, can be mimicked using artificial membranes (GelBond, Lonza Walkersville Inc., USA) for *in vitro* appressorial assays. The wild type is incapable of appressoria formation on non-inductive surfaces, but can do so only in the presence of exogenous cAMP or inhibitors of cAMP phosphodiesterases, suggesting that increased levels of cAMP may play a fundamental role in initiating early signaling events in appressorium formation [69]. Therefore, we asked whether elevated cAMP could influence precocious initiation of pathogenic development in the *pdeH*Δ. We quantified the efficiency of appressorium formation on inductive or non-inductive surface in the *pdeH*Δ, *pdeL*Δ and the wild type. The *pdeH*Δ elaborated appressoria on inductive (90±2.1%; Figure 4A and 4B) as well as on non-inductive surfaces (66±2.0%; Figure 4A and 4B), with a reasonably high frequency. In contrast, the wild type or the *pdeL*Δ conidia could form appressoria on inductive surface (75–80%), but failed to do so on non-inductive surface (4–7%; Figure 4A and 4B). Thus, the *pdeL*Δ behaves similar to the wild type and required proper inductive cues for pathogenic development. The two-celled conidia from the *pdeH*Δ *pdeL*Δ elaborated appressoria on inductive (Figure S3C) and non-inductive surfaces, however comparable quantifications were not possible due to the very low numbers of such structures formed. We thereby infer that the increased levels of intrinsic cAMP in the *pdeH*Δ are likely sufficient to uncouple surface dependency from pathogenic differentiation in *M. oryzae*.

Interestingly, we observed that a significant number of *pdeH*Δ conidia formed germ tubes that were extremely short or barely visible, prior to appressorium initiation. We scored and systematically classified the lengths of the germ tubes into three categories: short (short or barely visible), normal (equal to the length of the conidium) or long (longer than the conidium) prior to appressorium formation. We found that nearly 45±1.0% of the *pdeH*Δ conidia produced extremely short germ tubes prior to appressorium formation (Figure 4A; arrows), versus 25±1.8% in *pdeL*Δ strain and 13±1.7% in the wild type. Nearly 49±1.7% of the *pdeH*Δ conidia formed germ tubes of normal length, versus 69±2.3% in *pdeL*Δ and 74±2.2% in the wild type. Long germ tubes were seen in 6±1.7% of *pdeH*Δ compared to 6±2.0% and 13±1.8% in *pdeL*Δ and wild type conidia respectively. Based on the above results, we construe that PdeH influences surface sensing and guides germ tube growth during the early stages of infection-related morphogenesis in *M. oryzae*.

### PdeH negatively regulates appressorial development and maturation

**Figure 5:**
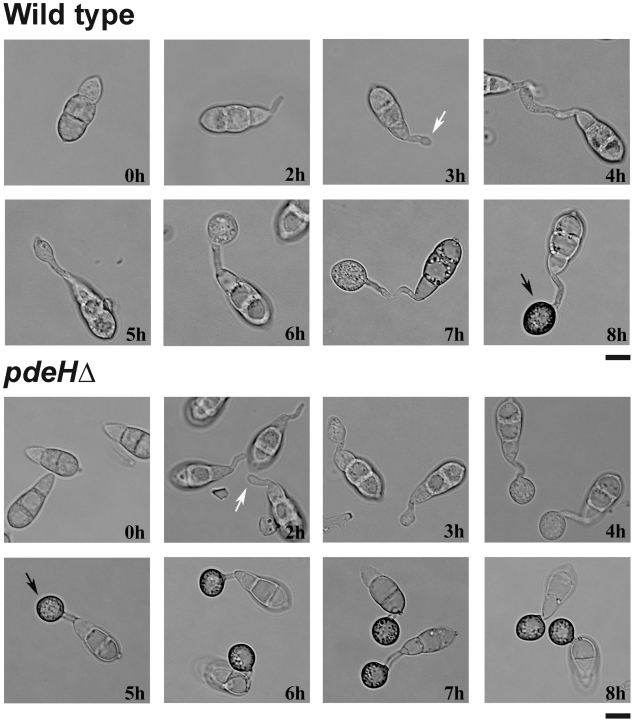
Loss of PdeH function advances appressorium formation and maturity. Comparative time-lapse observation of germination and appressorium formation in the wild-type or *pdeH*Δ conidia. Equivalent number of conidia from the indicated strains were inoculated on cover slips and incubated in a moist chamber at room temperature. The samples were analysed and micrographed every hour over an 8 h period. White arrows indicate the stage of appressorium initiation/germ tube hooking, whereas black arrows depict melanized appressoria. Scale bar = 10 micron.

We observed that the loss of PdeH derails cAMP-associated surface signaling and significantly accelerated appressorium formation. Time-course analysis revealed that *pdeH*Δ is significantly advanced in all stages of pathogenic development. On inductive surfaces, the wild type conidia underwent germ tube hooking at 3–4 hpi (Figure 5; white arrow), followed by tip swelling and growth into an immature appressoria by 5–6 hpi. By 8 hpi, the wild type formed melanized appressoria (Figure 5; black arrow). Under identical conditions, the *pdeH*Δ conidia initiated germ tube hooking as early as 2 hpi (Figure 5; white arrow), formed immature appressoria by 3–4 hours and finally melanized appressoria in about 5 hours (Figure 5; black arrow). Thus, the *pdeH*Δ not only initiated appressoria earlier but also formed melanized appressoria more rapidly, at least 3 h in advance compared to the wild type. The *pdeL*Δ behaved similar to the wild type taking about 8 hours to form melanized appressoria. Our findings suggest that precocious elevated cAMP levels can disrupt the temporal regulation of the processes that lead to proper initiation, development and maturation of appressoria in *M. oryzae*.

In *M. oryzae*, nuclear division or mitosis has been shown to precede appressorium formation, and arresting DNA replication or mitotic entry prevents appressorium formation [76]. Considering that a majority of *pdeH*Δ conidia precociously formed appressoria on inductive surfaces, we were interested in the mitotic status of the nuclei in *pdeH*Δ at different stages of pathogenic development. In wild type, mitosis generally occurred between 5–6 hpi, followed by migration of a daughter nucleus into the developing appressorium (Figure S4A). Interestingly, appressorial morphogenesis progressed rapidly and independent of nuclear division up to 3 hpi in the *pdeH*Δ conidia (Figure S4B; white arrows). By 4 hpi, mitosis generally occurred in the nucleus at the junction of the terminal cell of the conidium and the developing appressorium, followed by migration of a of the daughter nucleus into the maturing appressorium (Figure S4B; asterisk). We thus infer that elevated cAMP levels likely result in appressorial morphogenesis prior to nuclear division in the germinating *pdeH*Δ conidia.

### PdeH is required for pathogenesis in *M. oryzae*

**Figure 6:**
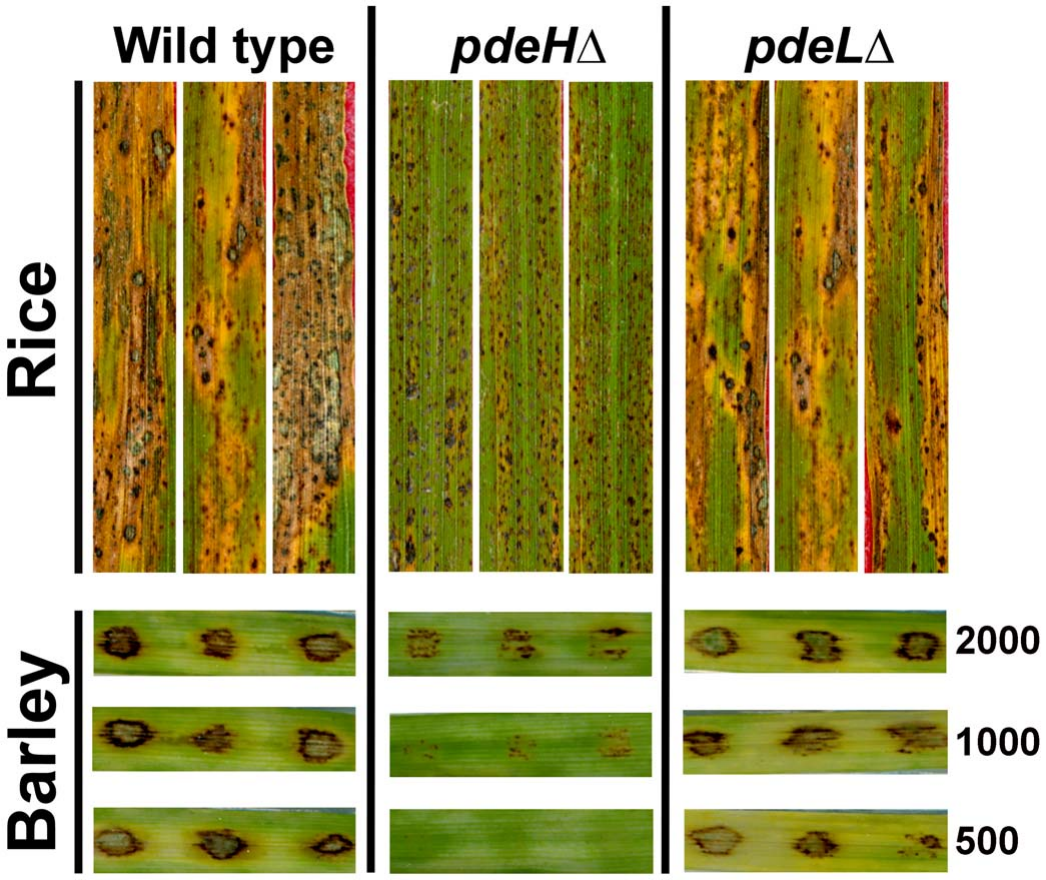
Loss of PdeH leads to reduced pathogenicity on host plants. Pathogenicity assays on barley leaf explants and rice seedlings. Rice seedlings (cultivar CO39) were spray inoculated with conidia from the wild type, *pdeH*Δ or *pdeL*Δ (as described in Materials and Methods) and disease symptoms scored after 9 d. Barley leaf explants were spot inoculated in triplicate with the specified number of conidia (per inoculation site) from the wild type, *pdeH*Δ or *pdeL*Δ and disease symptoms scored after 7 days.

In order to assess the ability to cause blast disease, we spray inoculated three-week old rice seedlings (variety CO39) with conidia from *pdeH*Δ or *pdeL*Δ strain. The wild type served as a positive control, and disease symptoms were evaluated nine days post inoculation. Rice seedlings inoculated with the wild type or *pdeL*Δ conidia showed numerous typical spindle-like, gray centered lesions that merged into one another. On the other hand, *pdeH*Δ conidia failed to infect the host efficiently and to cause typical blast lesions. Instead, the *pdeH*Δ formed minute brown speckles (Figure 6) that failed to coalesce and resembled the hypersensitive response (HR) seen on a moderately resistant host plant.

Barley leaf explants inoculated with various dilutions of wild-type or *pdeL*Δ conidia developed typical blast symptoms comprising spindle-shaped lesions with gray centers (Figure 6). Under similar conditions, *pdeH*Δ conidia failed to cause comparable disease symptoms at all conidial dilutions tested (Figure 6). Next, we tested if the aberrant structures formed by *pdeH*Δ *pdeL*Δ during the conidiation phase could cause disease on barley explants. Equivalent numbers of conidia from the wild type were used as control in parallel. The double deletion mutant failed to cause any disease symptoms on the inoculated leaves, unlike the wild type that displayed typical disease lesions (Figure S3B). The complemented *pdeH*Δ (RFP-PdeH expressing strain) was found to be as virulent as the wild type or the *pdeL*Δ on barley (Figure S2C).

Based on the disease assays on rice and barley, we conclude that PdeH (and by inference cAMP levels) is an important regulator of pathogenesis and disease severity during *M. oryzae* host interactions.

### The cAMP pathway regulates *in planta* growth and development of *M. oryzae*

**Figure 7:**
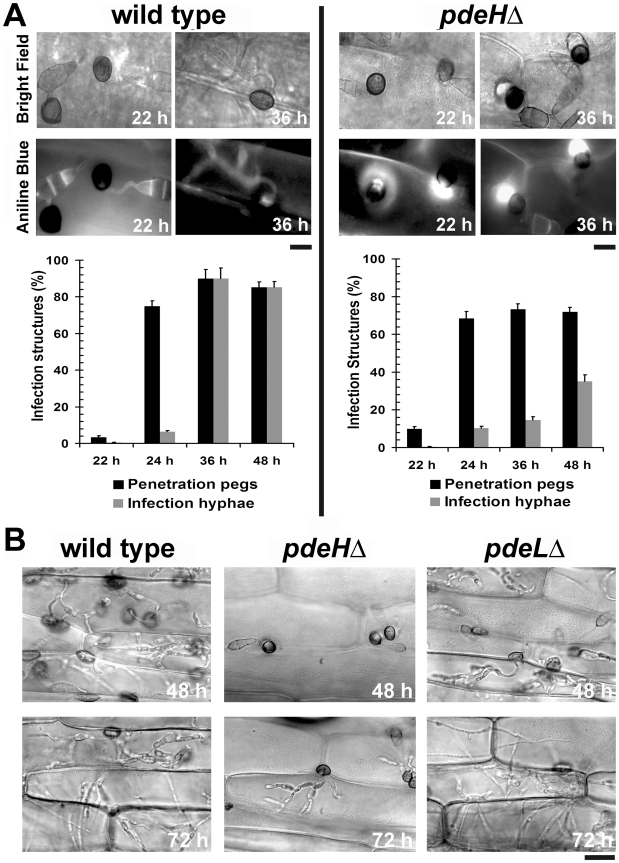
The *pdeH*Δ mutant is defective in its ability to colonize the host tissue. Analysis and quantification of host penetration and *in planta* development in the wild type and *pdeH*Δ. (**A**) Photomicrographs depicting aniline-blue stained host papillary callose deposits in the wild type or *pdeH*Δ at the specified time points post inoculation. Scale bar = 10 micron. Bar charts representing effective host penetration (black bars) as well as the efficiency of the subsequent development of infection hyphae (gray bars) in the indicated strains. (**B**) Microscopic observations tracking the development of infection hyphae and the ability to spread and colonize the host tissue. Micrographs depict *in planta* growth in the indicated strains, 48 and 72 hpi on barley leaf explants. Prior to microscopic observations, the inoculated leaf samples were clarified using methanol and stained with acid fuchsin. Scale bar = 25 micron.

Although, the *pdeH*Δ could efficiently form appressoria (∼90%) on rice and barley leaves, it failed to establish proper blast disease. Hence, we investigated the *in planta* development and quantified the host penetration ability of each strain (as judged by aniline blue staining for papillary callose deposits) of *pdeH*Δ in comparison to the wild type and *pdeL*Δ on barley leaves. At 22 hpi, only 3±0.8% of the wild type appressoria formed penetration pegs compared to 10±1.2% in case of the *pdeH*Δ strain, indicating a significant advancement (p<0.005) in the ability of the *pdeH*Δ appressoria to generate penetration pegs (Figure 7A). At 24 hpi, 75±2.8% of the wild type and 73±3.8% of *pdeH*Δ appressoria formed penetration pegs. As is evident from the graphs in Figure 7A, 90±5.0% of the wild type penetration pegs proceeded to form infection hyphae at 36 hpi. In contrast, only 14±1.7% of the penetration pegs developed into infection hyphae in *pdeH*Δ. These observations suggest that the *pdeH*Δ is not defective in host penetration, but shows severe reduction in differentiating infection hyphae. Further observations (48 hpi) revealed that 35±3.6% of *pdeH*Δ and 85±3.2% of the wild type penetration pegs advanced to form infection hyphae (Figure 7A). Furthermore, the *pdeH*Δ infection hyphae were significantly reduced in their *in planta* growth and colonization, with a majority being restricted to the first invaded cell (Figure 7B). In contrast, infection hyphae elaborated by the wild type achieved cross-wall penetration and spread. The *pdeL*Δ strain behaved in a manner similar to the wild type at all the time points tested (Figure 7B). The aberrant structures formed by *pdeH*Δ *pdeL*Δ during the conidiation phase failed to germinate and form appressoria even after 72 hpi, and as a consequence did not elicit any response from the host (Figure S3D). Interestingly, the two-celled conidia formed by the double deletion mutant successfully penetrated the host tissue as early as 22 hpi (Figure S3E). Further observations at 36, 48 and 72 hpi revealed that the double deletion mutant was blocked at the penetration stage, and failed completely to elaborate infection hyphae (Figure S3D and 3E).

These results indicate that the primary reason for the failure of the *pdeH*Δ to cause typical blast lesions is due to its compromised ability to form proper infection hyphae and to further advance its growth and spread in the host tissue. We thus hypothesize that PdeH-dependent regulation of cAMP signaling is key to successful host colonization by *M. oryzae*.

### Transcriptional regulation of *PDEH* and *PDEL*

**Figure 8:**
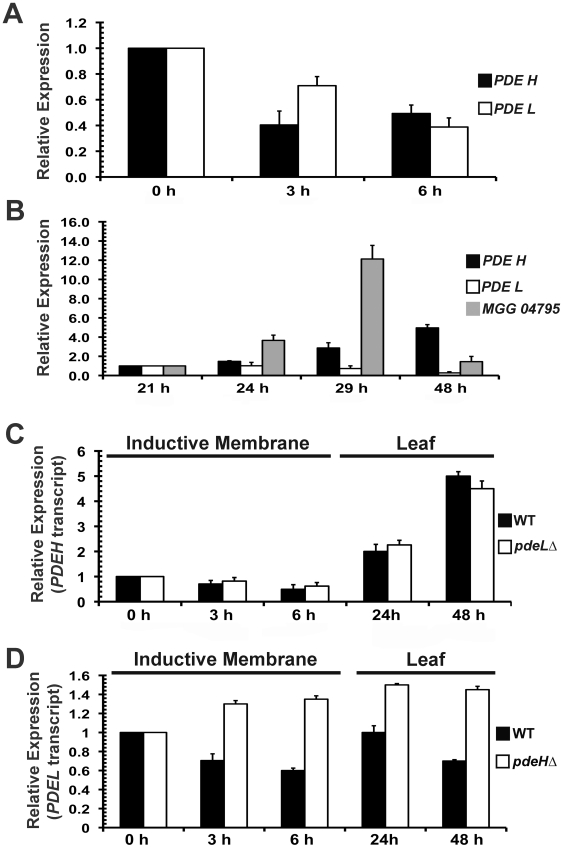
*PDEH* transcript is differentially regulated during infection-related development. Real Time RTPCR analysis and quantification of *PDEH* and *PDEL* transcript abundance in wild type and the indicated PDE mutants. (**A**) Total RNA from wild-type conidia inoculated on inductive Gelbond membrane for the indicated time points was subjected to Real-Time RTPCR-based quantification of *PDEH* (black bar) or *PDEL* (white bar) transcript. The expression levels at 0 h were considered as baseline and set as 1.0 (**B**) The levels of the *PDEH* (black bar) or *PDEL* (white bar) transcript was analyzed at the late stage of pathogenesis (24 h, 29 h and 48 h) in wild type *M. oryzae* inoculated on barley leaf explants. MGG_04795 was used as a positive control (gray bar). The expression levels at 21 h were set as 1.0 (**C**) Comparative quantitative analysis of *PDEH* transcript in the wild type (black bar) and *pdeL*Δ (white bar) using Real Time RTPCR at the specified time points. (**D**) *PDEL* transcript levels assessed in the wild type (black bar) and *pdeH*Δ (white bar) using Real Time RTPCR. In addition, the Real Time expression data was normalized to an endogenous control transcript (β-tubulin gene, MGG_00604). Each Real Time RTPCR reaction was repeated thrice independently with three biological replicates per data set.

In *M. oryzae*, cAMP signaling is known to regulate appressorium initiation and development [69], [71]. Extensive work carried out on PDEs in mammalian cells and in *Dictyostelium*, has described the existence of feedback loops (positive and negative) between varying cAMP levels and PDE gene expression [77], [78]. We were interested in elucidating if PDE transcripts were differentially regulated in response to fluctuating cAMP levels during pathogenic development in *M. oryzae*. Quantative Real-Time RTPCR analysis was used to measure the PDE transcript levels during different developmental stages during infection. The time points and tissues used were 0 h (freshly harvested conidia), 3 h (conidial germination and growth) and 6 h (appressorium initials). In addition, we also performed Real-Time RTPCR analysis on inoculated barley leaves at different stages of *in planta* growth. We included MGG_04795 (*BAS1*) as a positive control, since it has been shown to be highly upregulated during infection [79]. Comparative analysis of the Real-Time RTPCR data showed that *PDEH* transcript levels were significantly down regulated during the early stages of pathogenic development at 3 h (2.5 fold) and at 6 h (2 fold) compared to freshly harvested wild-type conidia (Figure 8A). A similar trend of reduction, 1.4 fold at 3 h and 2.5 fold at 6 h, was evident for the *PDEL* transcript. Compared to the 21 h time point, the *PDEH* transcript levels were consistently higher across all other time points tested: 1.5 fold at 24 h, 3 fold at 29 h and 5 fold at 48 h (Figure 8B). However, the levels of *PDEL* transcript did not show significant changes at similar time points. This suggests that the *PDEH* transcript levels are differentially regulated during early as well as the late stages of pathogenic differentiation in *M. oryzae*, likely in response to the varying cAMP levels at these stages of development.

### *PDEL* transcript is differentially regulated in the *pdeH*Δ mutant

We measured the levels of the *PDEL* transcript in *pdeH*Δ during pathogenic development. Conversely, the *PDEH* transcript levels were assessed in the *pdeL*Δ background. We used similar time points and fungal cell types as in the previous RTPCR experiment. *PDEH* transcript levels in the *pdeL*Δ were comparable to those in the wild type (Figure 8C and previous experiment). At the early stage, the *PDEH* transcript was down regulated in both the wild type and *pdeL*Δ strains (2 fold at 3 h and 2.5 fold at 6 h) and at the late stage, the *PDEH* transcript was upregulated: 2 fold at 24 h and 5 fold at 48 h (Figure 8C). However, compared to its levels in the wild type, the *PDEL* transcript were notably abundant in the *pdeH*Δ, during the early as well as the late stages of pathogenic development, across all time points and conditions tested (Figure 8D). We postulate that *PDEH* (and by inference PdeHp function) may be more responsive to even small changes in the levels of cAMP, whereas *PDEL* (and likely PdeL activity) likely responds to substantially elevated levels of cAMP, such as those prevalent in the *pdeH*Δ strain.

### Exogenous cAMP or IBMX simulates *pdeH*Δ-like defects in the wild type

**Figure 9:**
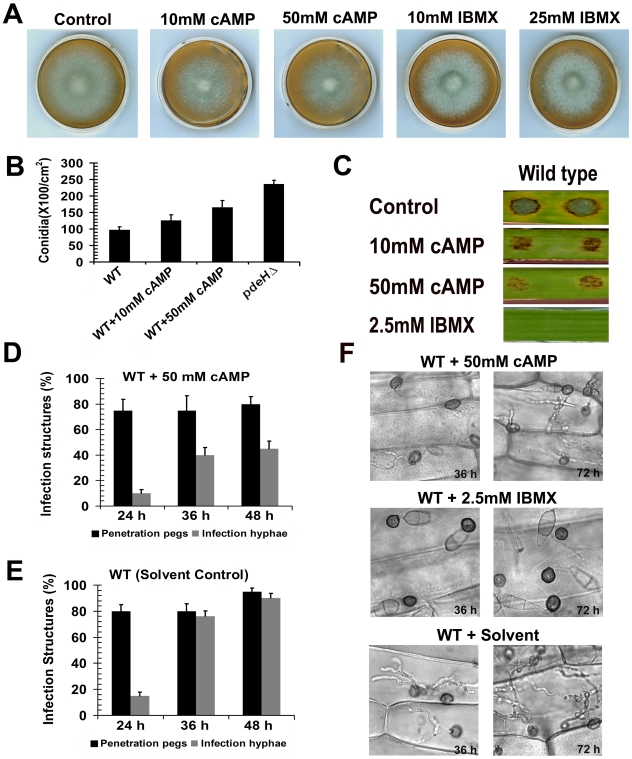
Exogenous addition of 8-Br-cAMP to the wild type results in *pdeH*Δ-like defects. Effects of exogenous 8-Br-cAMP or IBMX on wild type *M. oryzae*. (**A**) Colony morphology and phenotype of the wild type grown in the presence of 8-Br-cAMP or IBMX. Mycelial plugs from the wild-type *M. oryzae* colony were inoculated on PA medium containing the indicated amounts of 8-Br-cAMP or IBMX. These cultures were grown in the dark for 5 d prior to documentation. (**B**) Effect on conidiation. The wild type was grown in the presence of the indicated amounts of 8-Br-cAMP, and conidial numbers assessed after 6 d incubation under light. (**C**) Effect on lesion size and disease severity on barley leaf explants. The disease symptoms were scored 6 d after inoculation with wild type conidia in the presence of the specified amounts of 8-Br-cAMP (in water) or IBMX (in ethanol). (**D**) Effect of 8-Br-cAMP on infection structure development. Equal number of wild-type conidia was inoculated on barley leaf explants in the presence of 50 mM 8-Br-cAMP and the resultant penetration pegs (black bars) and infection hyphae (gray bars) quantified at the indicated time points. (**E**) Bar chart depicting the results of the control experiment performed in parallel, wherein wild type conidia inoculated on barley leaves in the presence of the appropriate solvent (ethanol). Penetration pegs (black bars) and infection hyphae (gray bars) were quantified at the indicated time points. (**F**) Excess cAMP or PDE inhibitor retards host colonization in *M. oryzae*. Barley leaf samples were inoculated with wild-type conidia in the presence of 50 mM 8-Br-cAMP or 2.5 mM IBMX and photomicrographed after incubation for the specified time intervals. Wild type treated with the equivalent amount of appropriate solvent (ethanol for IBMX) served as a parallel control.

Our results showed that the loss of PdeH function leads to higher basal levels of intracellular cAMP, thus affecting various aspects of asexual and pathogenic development. Therefore, we addressed whether exogenous addition of cAMP (8-Br-cAMP) or IBMX to the wild type would mimic the *pdeH*Δ defects. 8-Br-cAMP (a membrane permeable variant of cAMP) or IBMX (a phosphodiesterase inhibitor) have been extensively used in various studies to artificially cause the enhancement of endogenous cAMP levels [69], [71], [80], [81]. As shown in Figure 9A, wild type cultures grown in the presence of 8-Br-cAMP or IBMX showed a visible reduction in aerial hyphal growth, a defect reminiscent of the *pdeH*Δ strain.

We further explored if addition of 8-Br-cAMP to the wild type would enhance conidiation therein. Wild type cultures were initially grown in the dark for 2 d in the presence of 8-Br-cAMP and later exposed to constant illumination for a period of 7 d prior to quantification of conidia. An untreated wild type and *pdeH*Δ served as controls. Compared to the untreated control, wild type treated with 10 mM 8-Br-cAMP showed a marginal increase in conidial numbers; however 50 mM 8-Br-cAMP treatment evoked a 1.6 fold (p≤0.01) increase in conidia production (Figure 9B). This increase in conidiation although considerable, was not as high (2.3 fold; p<0.005) as exhibited by the *pdeH*Δ.

Furthermore, barley explants inoculated with wild-type conidia in the presence of 8-Br-cAMP showed a dose-dependent reduction in lesion size and lacked disease severity when compared to the control inoculations (Figure 9C). In contrast to the reduced lesion size and disease progression caused by 10 mM 8-Br-cAMP, the wild type conidia treated with 2.5 mM IBMX showed absolutely no disease symptoms on barley explants (Figure 9C). About 75±4.0% of 8-Br-cAMP-treated wild type appressoria successfully formed penetration pegs at 24 hpi; however by 36 hpi only 40±3.4% of the penetration pegs further developed into infection hyphae. By 48 hpi the number of infection hyphae formed increased to 47±3.0% (Figure 9D and 9F). At the corresponding time point, 90±3.6% penetration pegs formed by the control untreated sample had developed into infection hyphae (Figure 9E and 9F). IBMX-treated wild-type conidia formed appressoria efficiently on leaf tissues, but failed to elaborate penetration pegs even after 36 hpi. At 72 hpi, unlike the 8-Br-cAMP treated wild type, which elaborated infection hyphae, the IBMX-treated wild type failed to develop infection hyphae (Figure 9F).

Thus, 8-Br-cAMP- or IBMX-treated wild type significantly mimicked the *pdeH*Δ defects in aerial growth, conidiation, disease development and severity. These results support our previous experimental observations and our hypothesis that the defects exhibited by the *pdeH*Δ strain are indeed due to enhanced basal levels of cAMP, caused due to the loss of PdeH function in *M. oryzae*.

### Subcellular distribution of RFP-PdeH

We expressed an *RFP*-*PDEH* translational fusion construct in the *pdeH*Δ strain to track the subcellular localization and understand the spatial and temporal regulation of PdeH. This strategy was aimed at fluorescently tagging PdeH and to serve as a complementation tool that could potentially suppress the defects exhibited by *pdeH*Δ strain (Figure S2A and S2B).

Quantifications revealed that the number of conidiophores and conidia produced by the *RFP-PDEH* expressing *pdeH*Δ strain was comparable to the wild type (Comp *pdeH*Δ, Figure 2B). The *RFP-PDEH* strain lost the ability to elaborate appressoria on non-inductive surfaces. Infection assays showed that, unlike the *pdeH*Δ, the *RFP-PDEH* expressing strain had regained the ability to efficiently infect barley leaf explants at various spore concentrations tested (Figure S2C). These results indicate that the RFP-PdeH fusion protein was indeed functional and able to significantly suppress the *pdeH*Δ defects in conidiation and pathogenesis.

**Figure 10:**
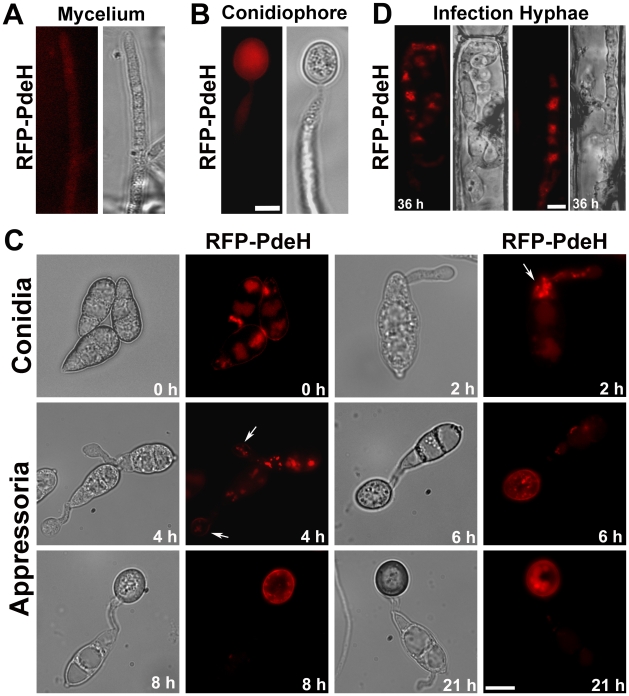
Subcellular distribution of RFP-PdeH fusion protein during different stages of pathogenic and asexual development. (**A**) Vegetative hyphae from RFP-PdeH strain were imaged after 3 d growth on PA medium. (**B**) Mycelial blocks of the RFP-PdeH strain were exposed to constant illumination on fresh agar medium, and developing aerial (conidiophore) structures imaged after 24 h. Scale Bar = 10 micron. (**C**) Conidia harvested from the RFP-PdeH strain were inoculated on plastic cover slips and incubated in a moist chamber prior to microscopic observations. Bright field and epifluorescence images were captured at the indicated time points, using the requisite filter sets. The arrows highlight the localization pattern of RFP-PdeH fusion protein at 2 hpi (punctate) and 4 hpi (plasma membrane). Scale Bar = 10 micron. (**D**) RFP-PdeH conidia were inoculated on rice leaf sheath and the infection hyphae imaged after 36 h using epifluorescence microscopy. Scale Bar = 10 micron.

The expression and localization of RFP-PdeH was then examined at different stages of asexual and pathogenic development. Vegetative hyphae and developing conidiophores showed a predominantly weak cytosolic distribution of RFP-PdeH (Figure 10A and 10B). Next, we looked at the distribution of RFP-PdeH at different stages of pathogenic development (Figure 10C). Conidia were harvested from the *RFP*-*PDEH* strain, inoculated on plastic cover slips and observed using epifluorescence microscopy. In freshly harvested conidia (0 h), RFP-PdeH localized as cytosolic punctae mostly in the terminal cell of the conidium. At 2 hpi, RFP-PdeH foci were predominant in the terminal cell as well as in the developing germ tube (Figure 10C; arrow). After 4 hpi, RFP-PdeH sustained its distinct punctate localization throughout the terminal cell of the conidium and the germ tube. Furthermore, there was a notable signal although weak, from the rim of the hooking germ tube, indicating a probable association with the plasma membrane (Figure 10C; arrow). The RFP-PdeH signal was weak and indiscernible in the conidia at 6 hpi and 8 hpi, but localized as randomly distributed punctae throughout the developing appressorium, and also showed a possibly weak association with the appressorial membrane. We excluded the possibility that the membrane localization was an artifact of melanization, since tricyclazole treated RFP-PdeH appressoria retained the weak association with the plasma membrane in addition to the distinct cytosolic punctae. (Figure S5A; arrows). In mature melanized appressoria (21 hpi; Figure 10C), the RFP-PdeH displayed a predominantly vacuolar localization. The RFP-PdeH was uniformly distributed through out the cytosol in the infection hyphae within the rice leaf sheath at 36 hpi (Figure 10D). Thus, RFP-PdeH displays a predominantly cytosolic distribution during asexual differentiation, whereas it localizes as distinct cytosolic foci, (and weakly to the plasma membrane of the germ tubes) during pathogenic development. RFP-PdeH was uniformly distributed throughout the cytosol in the infection hyphae during the biotrophic phase.

### Subcellular distribution of PRO*_Mpg1_*-GFP-PdeH and PRO*_Mpg1_*-GFP-PdeL

**Figure 11:**
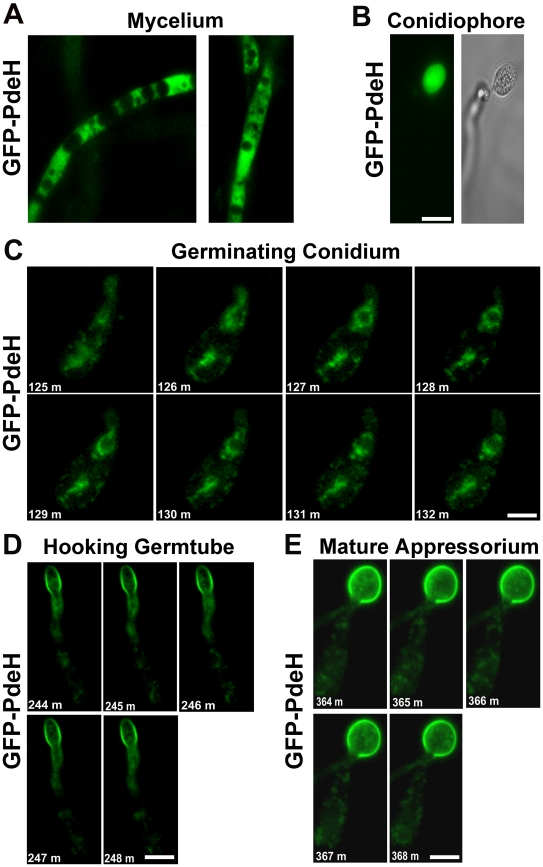
Localization and dynamic nature of the PRO*_Mpg1_*-GFP-PdeH during various stages of development in *M. oryzae*. (**A**) The PRO*_Mpg1_*-GFP-PdeH localized to the cytosol in the vegetative hyphae and (**B**) developing aerial structures (conidiophore). Scale Bar = 10 micron. (**C**), (**D**) and (**E**) Snapshots extracted from time-lapse movies (supplemental movies), showing the highly dynamic PRO*_Mpg1_*-GFP-PdeH, during different stages (specified sequentially) of pathogenic development. Scale Bar = 10 micron.

In order to better visualize the intracellular distribution and dynamics of PdeH, we expressed a *GFP*-*PDEH* translational fusion construct driven by the *MPG1* promoter [82] in the *pdeH*Δ strain. Similarly, we generated a strain expressing GFP-PdeL fusion protein under the *MPG1* promoter. We examined the expression and localization patterns of the PRO*_Mpg1_*-GFP-PdeH fusion protein during different stages of asexual and pathogenic development. Compared to the weak RFP-PdeH signal, the PRO*_Mpg1_*-GFP-PdeH showed a relatively strong cytoplasmic signal in the vegetative mycelia (Figure 11A). During asexual development, the GFP-PdeH fusion protein was predominantly cytosolic (Figure 11B), a pattern comparable to that displayed by the RFP-PdeH strain at a similar stage of development. To gain further insight into the dynamics, we made time-lapse observations of the PRO*_Mpg1_*-GFP-PdeH at different stages of pathogenic development encompassing the following time points: 2–3 hpi (conidial germination; Video S1 and Figure 11C), 4–5 hpi (hooking stage; Video S2 and Figure 11D), and 6–7 hpi (appressorium development; Video S3 and Figure 11E). Time-lapse analysis revealed that the GFP-PdeH fusion protein was associated with vesicular structures, which were highly dynamic and mobile (Video S1 and Figure 11C). Furthermore, co-staining with a nuclear dye (Hoechst 33342) confirmed a peri- and extra- nuclear localization of the GFP-PdeH foci (Figure S5B). At 4–5 hpi, GFP-PdeH localized to regions of the plasma membrane of the hooking germ tube in addition to being associated with highly mobile vesicles shuttling between the conidium and the germ tube (Video S2 and Figure 11D). In addition, GFP-PdeH was vesicular and enriched at the plasma membrane of the appressorium at 6 hpi (Video S3 and Figure 11E).

**Figure 12:**
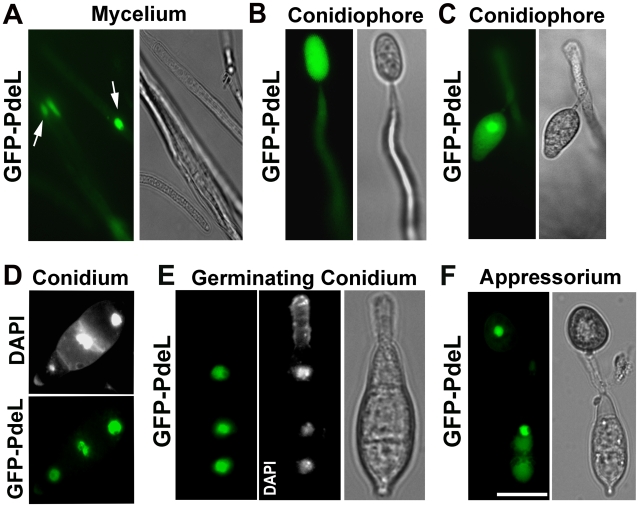
Predominant nuclear localization of the PRO*_Mpg1_*-GFP-PdeL during asexual and pathogenic development in *M. oryzae*. (**A**) Vegetative mycelia from a PRO*_Mpg1_*-GFP-PdeL colony, showing the nuclear distribution of GFP-PdeL (arrows). (**B**) and (**C**) GFP-PdeL in aerial structures such as an immature and a mature conidiophore respectively. (**D**) Conidia from PRO*_Mpg1_*-GFP-PdeL strain were stained with DAPI and assessed using epifluorescence microscopy. (**E**) A germinating conidium incubated on an inductive surface for 2 h, and stained with DAPI while (**F**) shows an epifluorescence microscopic image of GFP-PdeL in a mature appressorium formed after 12 h incubation on a plastic coverslip. Bright field images outline the fungal structures in each instance. Scale Bar = 10 micron.

Next, we examined the subcellular distribution of the PRO*_Mpg1_*-GFP-PdeL fusion protein in *M. oryzae*. Rather surprisingly, at all the stages of asexual and pathogenic development the GFP-PdeL localized predominantly to the nucleus (Figure 12A to 12F). The nuclear destination of GFP-PdeL was confirmed by co-staining with DAPI (Figure 12D and 12E). Thus, the predominant compartmentalization of PdeH within the cytosol (including the vesicular and plasma membrane localization) and of PdeL in the nucleus suggests that the high- and low-affinity PDEs differentially regulate and likely modulate distinct intracellular pools of cAMP. In summary, our results strongly suggest that appropriate and timely modulation of cAMP signaling, mainly by the PdeH, is critical for regulating the disease causing ability, as well as several important aspects of asexual development in *M. oryzae*.

## Discussion

Upon perception of light, the aerial hyphae of the rice-blast fungus *M. oryzae*, enter the asexual differentiation pathway to form conidiophores, which ultimately produce 3–4 conidia, each, in a sympodial manner [65], [67], [83], [84]. The pattern and number of conidia produced per conidiophore is highly regulated and important for proper conidial function [67], [71], [84]. Heterotrimeric G proteins and cAMP signaling have been shown to be important regulators of asexual development in *M. oryzae* [71], [85], [86] and several other filamentous fungi such as *Aspergillus*, *Cryphonectria*, *Neurospora* [44], [87], [88], [89], [90], [91]. The cAMP signaling is also a key regulator of the pathogenic differentiation in *M. oryzae* [71], [81], [85], [92], [93]. The cAMP PDEs constitute a large family of enzymes that hydrolyse cAMP and provide the sole means of inactivating this important second messenger in eukaryotic cells. In addition, PDEs function to regulate the specificity, intensity and temporal duration of cAMP signaling [2], [4], [94], [95], [96], [97], [98].

While a functional role for the high- and low-affinity PDEs in filamentous fungi such as *Neurospora* or *Aspergillus* has not yet been defined, our preliminary analysis does indicate that these fungi do possess PDE orthologs (Figure 1A and 1B) with the conserved consensus sequences typical of both the variants. However, specific roles have been defined for cAMP signaling in *C. albicans* and *C. neoformans*, representing the dimorphic or pseudo-filamentous fungi. In addition to regulating the development of hyphal cell wall and membrane structures, the high-affinity PDE in *C. albicans* has been shown to regulate intracellular levels of cAMP during stress conditions and virulence. However, a role for the low-affinity PDE has not yet been established [23], [24], [25], [27], [61], [99]. Rather surprisingly, a reversal in function has been suggested for the PDEs in *C. neoformans*, wherein the low-affinity PDE modulates the cAMP signaling [30]. In the present study, we investigated the implications of PDE-based regulation of the intracellular cAMP during asexual and pathogenic development in *M. oryzae*. We analyzed in detail the effects of gene-deletion of the two cAMP phosphodiesterase-encoding genes *PDEH* and *PDEL* (either independently or in combination). Our data suggests unique and non-redundant roles/functions for PdeH and PdeL; but underscores the overall importance of PdeH as a critical regulator of cAMP signaling in *M. oryzae*. Furthermore, PdeH and PdeL appear to be differentially compartmentalized: PdeL being predominantly nuclear while PdeH is largely cytosolic but confined to perinuclear regions as vesicular foci during conidial germination and to the developing appressorial membrane during the later stages of infection-related morphogenesis. Such an association of a PDE to membranous regions around the nucleus, has been demonstrated through fractionation studies in yeast [100].

Unlike PdeL, which we found to be dispensable for asexual development and virulence in *M. oryzae*, PdeH was a key regulator of cAMP levels during these two important phases of growth. This suggests that PdeH is able to compensate for the loss of PdeL. However, simultaneous loss of both PDEs was deleterious to proper growth and asexual development, suggesting that above a particular threshold, excess cAMP is detrimental.

Our results further suggest that PdeH regulates cAMP signaling at two critical steps during *M. oryzae* pathogenesis namely: infection-structure formation and host invasion. This hypothesis is based on the direct assessment of cAMP levels, at the initial (increased) and final (decreased) stages of infection in the wild type and the *PDE* mutant strains of *M. oryzae*. An initial increase in cAMP [69], [71] is a likely consequence of a concomitant decrease in *PDEH* transcript levels during the early phase of appressorium formation. Such a decrease in PDE function could also be a consequence of post-translational modifications (as demonstrated in yeast and various mammalian isoforms [2], [12], [30], [54], [94]) and/or the dynamic recycling of the PdeH-containing multivesicular foci away from the active growth or signaling zones in the germ tube tips. Nonetheless, the down-regulation of cAMP at appressorium maturity is extremely important since it has a strong bearing on successful colonization of host tissue by *M. oryzae*. We demonstrate that higher levels of exogenous cAMP retard *in planta* development in the wild type at the late stages of infection, a phenotype reminiscent of the loss of PdeH function during rice-blast disease. On the contrary, increased intracellular cAMP levels (*pdeH*Δ or exogenous addition) enhance asexual development in *M. oryzae*. However, there appears to be a threshold and dose-dependent response to high cAMP, since loss of both PDEs (simultaneous) led to aberrant fungal structures and a complete cessation of proper conidiation. The genetic data and biochemical analysis presented here support the model that PdeH (like Rgs1, Regulator of G protein Signaling [71]), negatively affects the cAMP signaling in *M. oryzae*.

We show that overall cAMP levels are not significantly affected in the *pdeL*Δ either during conidia or appressoria formation, and that PdeH is the major hydrolyzing enzyme to control the baseline levels of cAMP in these important cell types. However, taking into consideration the phenotype and the elevated cAMP levels in the double deletion mutant, it is possible that PdeL gains importance under conditions when the intracellular levels of cAMP are very high, like in the *pdeH*Δ strain. We propose that PdeH may not be directly involved in cell shape and morphogenesis, but that the *pdeH*Δ phenotype is brought about by the inappropriate or untimely activation of signaling through cAMP accumulation. For instance, majority of the *pdeH*Δ appressoria were initiated without any discernable germ tube emergence or extension from the terminal cells of the conidia. It is tempting to speculate that cAMP signaling is involved in cessation of germ tube growth prior to initiating the hooking stage important for appressorium initiation. Our preliminary data (Figure S4) also suggests that increased cAMP levels likely override the requirement for nuclear division prior to initiation of appressoria. However, additional experiments are required to further investigate the relevance of the *pdeH*Δ defects in understanding infection-related morphogenesis in conjunction with cell cycle progression in the rice-blast fungus. It is plausible that PdeL activity likely responds to enriched gradients or pools of cAMP present in the nucleus, as has been suggested by Huston.E *et al* [101].

Furthermore, the principles of compartmentalized cAMP signaling may well apply to highly polarized cell types in *M. oryzae*, namely conidia, appressoria and infection hyphae, wherein relevant changes in cAMP levels may be controlled in space and time and could be anchored within subcellular compartments such as the nucleus or the outer membranes. It is also possible that the *M. oryzae* adenylate cyclase (mac1) [72], does not produce cAMP constitutively but only in small bursts confined to the active growth and signaling zones, in response to inductive stimuli.

Our data indicates that cell signaling in *M. oryzae* responds to rapid albeit small changes in cAMP levels, since loss of PdeH leads to increased accumulation of intracellular cAMP. Furthermore, our findings clearly establish that the overall modulation of the non-nuclear cytosolic pools of cAMP by the high-affinity phosphodiesterase at two distinct phases (up-regulation during early stages and down-regulation during the late stages) is vital for proper pathogenic and infectious development in *M. oryzae*. We do not rule out dynamic fluctuations (albeit minor) of cAMP within these two major stages of pathogenic differentiation. Furthermore, it is possible that the reduced *in planta* growth of the *pdeH*Δ in *M. oryzae* is a likely consequence of the increased sensitivity towards stress conditions encountered within the host plant. For instance, the cAMP pathway is a major regulator of stress signaling in *Candida* and the *pde2*Δ mutant is more sensitive to stress, particularly peroxide and cadmium [99]. Future experiments would address the issues related to associations (if any) between stress signaling and cAMP levels in *M. oryzae*.

## Materials and Methods

### Fungal strains and growth conditions

The wild type *M.oryzae* strain, B157, was obtained from the Directorate of Rice Research (Hyderabad, India). *M. oryzae* strains were cultured either on Prune agar medium at 28°C (PA; per liter: 40 mL prune juice, 2.5 g lactose, 2.5 g sucrose, 1 g yeast extract, and 20 g agar) or Complete medium (CM; per liter: 0.6% yeast extract, 0.6% casein hydrolysate, and 1% sucrose). Either CM agar (for hygromycin selection) or Basal Medium (BM; per liter: Yeast nitrogen base without amino acids 1.6 g, asparagine 2 g, glucose 10 g, NH_4_ NO_3_ 1 g, and 20 g of agar, for ammonium glufosinate selection) was used for the selection of fungal transformants. To assess the growth and colony characteristics, wild type as well as the deletion strains were cultivated on PA medium at 28°C for one week. For quantitative analysis of conidiation, fungal strains were cultivated on PA medium in the dark for a day, followed by incubation under constant illumination for 7 d at room temperature. Mycelia used for genomic DNA or total RNA extraction were harvested from cultures grown in liquid for 2–3 days at 28°C as described [102].

### Appressorial assays and pathogenicity tests

Conidia were harvested by scraping the surface of the colonies with inoculation loops in the presence of sterile water, and the fungal biomass collected in Falcon tubes (BD Biosciences, USA). The suspension was vortexed for a minute to ensure complete detachment of conidia from the mycelia, and then filtered through two layers of Miracloth (Calbiochem, San Diego, USA). The conidia were pelleted by centrifugation at 3000 rpm for 15 minutes at room temperature. The conidial pellet thus obtained was washed twice and re-suspended in sterile water. The radius of the colony was initially measured to calculate the surface area of the colony. Conidia produced by a given colony were quantified using a hemocytometer and reported as the total number of conidia present per unit area of the colony.

For appressorial assays, the harvested conidia were re-suspended at 10^5^ conidia per mL in sterile water. Droplets (20 µl) of conidial suspension were placed on plastic cover slips or hydrophilic side of GelBond membrane (Lonza Walkersville Inc., USA) and incubated under humid conditions at room temperature. The total number of appressoria was quantified after 16 h. Microscopic observations were made using an Olympus BX51 epifluorescence compound microscope with bright field optics. For pathogenicity assays and assessment of blast lesions, a dilution series of the conidial suspension was inoculated on detached barley leaves, and incubated for 7–9 days in a growth chamber (22°C, 16 h light/8 h dark). Spray inoculations on rice cultivars were conducted as previously described [103]. For host penetration assays, conidial suspensions in sterile water were inoculated on barley leaf explants and assessed after 22 h, 24 h, 36 h and 48 h. Penetration pegs and infection hyphae were detected by staining for papillary callose deposits using Aniline blue[104]. Fungal structures were stained with acid fuchsin as described [74]. Given their lack of sufficient numbers, we could not carry out spray inoculations of the rice seedlings with the aberrant structures formed by the double deletion mutant (during the conidiation phase). However barley infection assays were carried out with *pdeH*Δ *pdeL*Δ with a conidial load normalized to at least 50 two-celled conidia-like structures per droplet. A parallel wild type control with equivalent conidial load was included.

The cyclic AMP analog, 8-Br-cAMP (BioLog, Germany) was first added at 0 h and again supplemented after 20 hpi (a final concentration of 10 mM or 50 mM). Stock solutions of 8-Br-cAMP (100 mM) were made in water, while IBMX (25 mM) (Sigma Aldrich, USA) was dissolved in 99% ethanol [71], [80]. For tricyclazole (Cluzeau Info Labo, France) treatment, conidia from the requisite strain were inoculated on cover slips in the presence of 8 µg/mL tricyclazole [105]. Nuclear staining was carried out using DAPI (diamidino-2-phenylindole; Sigma Aldrich, USA) essentially as described [106], although with minor modifications to suit *M. oryzae* conidia or germlings. Freshly harvested conidia were appropriately diluted and inoculated on hydrophobic plastic cover slips in a moist chamber at room temperature (RT) for 1–2 hours. The cover slips with the adherent germlings were then fixed with formaldehyde (3.7% final concentration), for 5–10 minutes at RT. The fixed samples were then washed gently with distilled water, prior to treatment with Triton X-100 (0.1% final concentration) for 1 minute at RT. The samples were washed thrice with distilled water, and finally incubated in the dark for 10–15 minutes, with a solution of DAPI dissolved in water at a final concentration of 1 µg/ml, and visualized using the Olympus BX51 epifluorescence microscope. Nuclear staining during live imaging of GFP-PdeH was achieved using Hoechst 33342 (Sigma Aldrich, USA) at a concentration of 1 µg/ml (in water) for 20 minutes. Cell wall and septa were stained using calcofluor white in water at a final concentration of 10 µg/ml. Samples were washed twice with sterile distilled water prior to visualization by epifluorescence imaging using the Olympus IX71 microscope.

### Nucleic acid related methods

Standard molecular manipulations were performed as described [107]. Fungal genomic DNA was extracted using Master Pure Yeast DNA purification kit (Epicenter Biotechnologies). Plasmid DNA was isolated with Geneaid High Speed Plasmid Mini kits. Homology searches of DNA/protein sequences were performed using the BLAST program [108] and multiple sequence alignments carried out with ClustalW [109] and Boxshade (http://bioweb.pasteur.fr/seqanal/interfaces/boxshade.html).

### Gene deletion and complementation analysis

Deletion mutants of *PDEH* (NCBI accession XP_360290) or *PDEL* (NCBI accession XP_367803) were generated using the standard one-step gene replacement strategy. Genomic DNA fragments (about 1 kb each) representing the 5′ and 3′ UTR of *PDEH* (MGG_05665) gene were amplified by PCR, ligated sequentially to flank the hygromycin phosphotransferase gene (*HPH1*) cassette, in pFGL44, to obtain pFGLpdeHKO. The following primers were used to amplify the 5′and 3′ UTR of the *PDEH* gene: PDEH-5F (5′- CAGAGAGAATTCAGCACCAGCATGGCACCACTATC), PDEH-5R (5′- CAGAGATCTAGACAAAGAGCGTCCAGTCATAAGACT), PDEH-3F (5′- CAGAGACTGCAGGTTCAGTACTACTGTTCACTCAGAT), PDEH-3R (5′- CAGAGAAAGCTTACGCATTACCCAATGTTGGCATC). pFGLpdeLKO was obtained by amplifying approximately one kb fragments of genomic DNA corresponding to the 5′ and 3′ UTR of *PDEL* (MGG_07707). The PCR fragments obtained were cloned in pFGL97 to flank the bialaphos resistance gene cassette (*BAR*). The 5′ UTR was amplified using the following primer pairs: PDEL-5F (5′CAGAGAGGTACCCCGTTTGCTACCTGTGGCCAACG), PDEL-5R (5′- CAGAGAGGATCCCCGCCCGTCCCGTCTAGCCCAGCTGGGCT); The 3′ UTR was amplified using the following primer pairs PDEL-3F (5′- CAGAGACTGCAGTGCGACCCTATGACAGTCCCCT), PDEL-3R (5′- CAGAGAAAGCTTGAGGCCGCCAATGCCACGAGCGC). Underlined text in the primer sequences represents the restriction enzyme sites used for cloning purposes. The sequences of pFGLpdeHKO as well as the pFGLpdeLKO gene replacement constructs was confirmed and the plasmids were introduced into wild type B157 strain via *Agrobacterium* T-DNA-mediated transformation to specifically replace the *PDEH* or *PDEL* genes with *HPH1* or *BAR* respectively. Resistance to hygromycin (CM containing 250 µg/ml hygromycin, A.G.Scientific Inc, USA) or ammonium glufosinate (BM containing 40 µg/ml ammonium glufosinate, Cluzeau Info Labo, France) was used to select the fungal transformants. Southern blot analysis and PCR were performed to identify the correct gene-replacement events (*pdeH* **::** *HPH1* or *pdeL* **::** *BAR*). The double mutant *pdeH*Δ *pdeL*Δ was like wise generated by introducing pFGLpdeHKO into the *pdeL*Δ strain. Southern blot analysis was used to confirm the gene replacement (*pdeH* **::** *HPH1*) and copy number of the integron.

### Plasmid constructs for RFP-*PDEH* fusion

To generate an in-frame translational fusion of RFP-PdeH, the promoter fragment (about 1 kb) of the *PDEH* gene was PCR amplified from genomic DNA of the wild type, using the primers (5′- CAGAGAGAATTCGTTCGGCTCAATTCAATTCGA) and (5′- CAGAGAGAGCTCCGTGGGCCCAAAGAGCGTCCA). The RFP coding sequence was amplified from pDsRED-Monomeric-N1 (Clontech, CA, USA) using the primers (5′- CAGAGAGCGCTCATGGACAACACCGAGGAC) and (5′- CAGAGACCATGGCCTGGGAGCCGGAGTG). A 3.9 kb fragment comprising the entire *PDEH* coding sequence as well as the downstream 1 kb region was amplified using the primers (5′- CAGAGACCATGGAGAATGCTGCCTGCAAT) and (5′-AGAGAGGATCCTGCCAAGTTGTCACTTTCCAAGT). Underlined text in the primers corresponds to the restriction enzyme sites used for cloning. All the three PCR products obtained were cloned into pFGL97 to get pFGL-NT-Comp construct with resistance to ammonium-gluphosinate (Cluzeau Info Labo, France) as a fungal selectable marker. This construct was introduced as a single-copy insertion into the *pdeH*Δ strain.

### Plasmid constructs for PRO*_Mpg1_*-GFP-*PDEH* and PRO*_Mpg1_*-GFP-*PDEL* fusion

eGFP was amplified using the following primers (5′-CAGACCATGGTGAGCAAGGGCGAGGA) and (5′- CAGACATATGCTTGTACAGCTCGTCCAT) from pEGFP-N1 (Clontech, CA, USA). A ∼3.0 kb fragment encoding the complete *PDEH* coding sequence was amplified using the primers (5′- CAGACATATGGAGAATGCTGCCTGCAAT) and (5′- CAGATCTAGATCAACCAGCAGTGTC). Similarly, a ∼2.6 kb fragment coding for the entire *PDEL* sequence was amplified using the following primer pairs (5′- CAGACATATGGGCGAGGGCAGCGCCGAA) and (5′- CAGATCTAGATCACAAGTACAATGCCTCGCCA). Underlined text denotes the restriction enzyme sites used for cloning. In both the cases the amplified PCR products (eGFP and *PDEH*) or (eGFP and *PDEL*) were cloned in-frame, under the constitutive *MPG1* promoter and *TrpC* terminator in pFGL275 to obtain pFGL-PRO*_Mpg1_*-GFP-*PDEH* and pFGL- PRO*_Mpg1_*-GFP-*PDEL* constructs respectively. pFGL-PRO*_Mpg1_*-GFP-*PDEH* was introduced into the *pdeH*Δ strain, while the pFGL-PRO*_Mpg1_*-GFP-*PDEL* was introduced into the wild type strain via *Agrobacterium* T-DNA-mediated transformation. Fungal transformants was selected based on resistance towards ammonium glufosinate (BM containing 40 µg/ml ammonium glufosinate, Cluzeau Info Labo, France). Transformants were screened for GFP expression and confirmed by sequencing genomic DNA and southern blot analysis.

### Quantitative Real-Time RT-PCR

Total RNA was extracted using RNeasy Plant Mini Kit (Qiagen, USA) from ungerminated wild type conidia (0 h), or WT conidia germinated for 3 h and 6 h on hydrophobic surface of GelBond membranes (Lonza Walkersville Inc., USA). Total RNA was also extracted from wild type conidia inoculated on detached barley leaves at 21 h, 24 h, 29 h and 48 h. Purified RNA was treated with DNase (Roche Diagnostics, Germany) and were verified as DNA free by using them directly as template in a PCR assay. First-strand cDNA was then synthesized using 2 µg total RNA and AMV Reverse Transcriptase (Roche Diagnostics, Germany) in the presence of oligo dT_18_. Reactions were performed in 10 µl volume containing 25 ng of cDNA, 0.5 µM of each primer and 5 µl of Power SYBR Green PCR Master Mix (Applied Biosystems) using 7900HT Fast Real-Time PCR system. The following cycling parameters were used, 50°C 2 minute, 1 cycle; 95°C 10 minute, 1 cycle; 95°C 15 second, 60°C 1 minute, 40 cycles. Relative abundance of transcripts was analyzed by the 2^−ΔΔCt^ method [110] and average threshold cycle (Ct) normalized to β-tubulin transcript for each condition as 2^−ΔCt^. Fold changes were calculated as 2^−ΔΔCt^_._ The primer pairs used for quantitative RTPCR were: For MGG_04795 (5′- TTTGATCAGCGTTACCAAGG) and (5′- CGGTGACCAACATTCTCTTG); MGG_00604 (5′- CATGATGGCTGCTTCTGACT) and (5′-CGACGAGTTCTTGTTCTGGA) MGG_ 05664 (5′- GCTTGAGCGCTGGAGAATGT) and (5′- TAACGAGCCGATCTGTACCA); MGG_07707 (5′- CTTCTACAGCAGAGACACC) and (5′- GCTCCTGCATAATAATGTCC). Each Real-Time RTPCR reaction was repeated three times independently with three biological replicates per sample. The melting curve analysis was used to determine the specificity of the amplifications. Reactions with no cDNA added (no template controls) were performed in parallel and monitored for primer dimers. Real-Time RTPCR primers were designed to span introns whenever possible.

### Quantification of intracellular cAMP

Quantification of intracellular levels of cAMP was essentially carried out as described [71]. Freshly harvested conidia (0 h), conidia germinated for 6 h, as well as conidia that have formed mature appressoria following 21 h, 24 h, 28 h or 30 h incubation on the inductive (hydrophobic) surface of GelBond membranes (Lonza Walkersville Inc., USA) were harvested, frozen and lyophilized for 16 h. Lyophilized fungal biomass was individually ground into a fine powder in liquid nitrogen and equal weights of the fine powder was re-suspended in 200 µl of chilled 6% Trichloro acetic acid (TCA) and incubated on ice for 10 min. After centrifugation at 4000 rpm for 15 min at 4°C, the supernatant was collected and extracted four times with five volumes of water-saturated diethyl ether to remove the TCA. The remaining aqueous extract was lyophilized and dissolved in the assay buffer. In total, each assay was repeated three times independently with two biological replicates for each strain. The cAMP levels were quantified using the cAMP Biotrak Immuno-assay System (Amersham Biosciences, USA). For estimation of cAMP levels, the *pdeH*Δ *pdeL*Δ mutant was grown on nitrocellulose membranes (Millipore, USA) placed on prune agar (PA) medium. These cultures were incubated at 28°C for 3 d, followed by exposure to light for 24 h. The resultant colonies were scraped gently in the presence of diluted assay buffer to harvest the mycelial and aerial growth. Care was taken not to damage or include the nitrocellulose membrane in the fungal biomass. Further processing of the sample for cAMP measurements, was essentially carried out as described above. The double deletion mutant did not display any difference in growth pattern or characteristics on the nitrocellulose membrane, compared to growth without such membranes under the same conditions.

### Microscopic analysis

Bright field and epifluorescence microscopy utilized the Olympus IX71 or BX51 (Olympus, Japan) equipped with a Plan APO 100X/1.45 or UPlan FLN 60X/1.25 objective with appropriate filter sets. Images were captured using a Cool SNAP HQ camera (Photometrics, USA) and processed using Image J (National Institutes of Health, USA), MetaVue (Universal Imaging, USA) and Adobe Photoshop 7.0 (Adobe Inc, USA).

## Supporting Information

Figure S1 Schematic representation and deletion analysis of PDE genes in *Magnaporthe oryzae*. (A) Diagrammatic presentation of the *ScPDE2* ortholog *MGG_05664* in *M. oryzae*. Solid bars and short open boxes represent coding regions and introns (respectively), while dashed lines indicate the genomic flanks used for gene targeting. Restriction enzyme sites used for cloning are depicted (E: *Eco*RI, X: XbaI, P: *Pst*I and H: *Hind*III) and *HPH1* refers to the hygromycin-resistance gene cassette used for gene replacement. Scale bar equals 1 kb and delineates the probe used for Southern blot analysis. (B) Diagrammatic representation of the *ScPDE1* ortholog *MGG_07707* in *M. oryzae*. The solid bars indicate exons while open boxes depict introns. Dashed lines indicate the flanks used for targeted gene disruption. K: *Kpn*I, B: *BamH*I, P: *Pst*I and H: *Hind*III are the restriction enzyme sites used for cloning, and *BAR* refers to the bialaphos-resistance gene cassette. Scale bar represents 1 kb and the probe used for Southern analysis. (C) Southern analysis for confirmation of the *PDEH* deletion strains. Genomic DNA from the wild type or *pdeH*Δ was digested with *Xho*I and probed with a 1 kb fragment representing the 3′UTR region. The appearance of a 5.7 kb fragment in the deletion strain (lane 1) and a 2.7 kb in the control wild type (lane 2), indicated an accurate *PDEH* replacement event. The blot was stripped and was re-probed with *HPH1* to detect the diagnostic 5.7 kb band in the *pdeH*Δ. (D) DNA gel blot analysis to confirm the *PDEL* deletion. *Nco*I-digested genomic DNA extracted from wild type or *pdeL*Δ was probed with the PDEL 3′UTR fragment. Presence of the 4.3 kb fragment in the mutant (lane 1) and a 1.8 kb fragment in the wild type (lane 2), indicates precise *PDEL* replacement. The membrane was re-probed with *BAR* to further confirm the diagnostic 4.3 kb fragment in the *pdeL*Δ. (E) Confirmation of the *pdeH*Δ *pdeL*Δ strain. The double deletion mutant was created by deleting the *PDEH* gene in the *pdeL*Δ background. Confirmation of the requisite gene replacement (*pdeH::HPH1*) was carried out as described in (C) above. The estimated size (in kb) of the relevant fragments is indicated.
(2.68 MB TIF)

Figure S2 Genetic complementation analysis of the *pdeH*Δ strain. (A) Southern blot analysis of the RFP-PdeH expressing *pdeH*Δ strain. The RFP-PdeH expressing *pdeH*Δ strain was generated by transforming an in-frame *RFP-PDEH* translational fusion construct to integrate in the *pdeH*Δ background. *Nco*I digested genomic DNA from the RFP-PdeH expressing *pdeH*Δ (lane 1) or the wild type (lane 2) was subjected to Southern blotting with an RFP specific probe. Copy number of the integron (complementation cassette) was judged by southern analysis. The size of the relevant fragment in kilo base-pair is depicted. (B) Growth and colony characteristics of the RFP-PdeH expressing *pdeH*Δ strain (upper panel) grown for a week in the dark on prune agar medium. The lower panel shows a medial cross section of the above colony depicting significant restoration of aerial hyphal growth. (C) The RFP-PdeH expressing *pdeH*Δ regained the ability to cause blast disease. Barley leaf explants were inoculated with indicated number of conidia (in triplicate) from the wild type or the RFP-PdeH expressing *pdeH*Δ. The lesions formed were scored 7 d post inoculation. The RFP-PdeH expressing *pdeH*Δ formed typical disease lesions comparable to the wild type at the respective conidial dilutions tested.
(6.58 MB TIF)

Figure S3 Characterization of the *pdeH*Δ *pdeL*Δ mutant. (A) Conidiation is severely reduced upon loss of PDE genes. Close up view of the surface of the *pdeH*Δ *pdeL*Δ colony showing aberrant conidiation-related aerial structures (arrows). Inset depicts the rare conidia-like structure formed by the *pdeH*Δ *pdeL*Δ mutant. Scale bar = 10 micron. (B) Barley leaf explants inoculated with aberrant conidiation structures from *pdeH*Δ *pdeL*Δ or conidia from the wild type (WT) were analyzed 7 dpi. (C) Conidia from the wild type or double deletion mutant were inoculated on coverslips for 24 h and stained with calcofluor white prior to epifluorescence imaging. The arrow highlights the single septa in the conidia formed by the double deletion mutant. Scale bar = 10 micron. (D) The aberrant structures formed during the conidiation phase in the *pdeH*Δ *pdeL*Δ do not elicit a host response, unlike the two celled conidia. Photomicrographs depicting aniline-blue stained host papillary callose deposits in the *pdeH*Δ *pdeL*Δ strain at the indicated time points post inoculation. Scale bar = 10 micron. (E) Bar chart representing effective host penetration (black bars) as well as the elaboration of infection hyphae (gray bars) by the two celled conidia (n = 25) in the double deletion mutant. However the resultant penetration fails to elaborate/develop infection hyphae in the host. Asterisk highlights the solitary infection hypha detected at 48 h. Values represent mean ± S.E from two independent experiments involving 25 conidia per sample.
(8.01 MB TIF)

Figure S4 Analysis of nuclear division during appressorial morphogenesis in *M. oryzae*. (A) and (B) conidia from the wild or the *pdeH*Δ were inoculated on plastic cover slips in a moist chamber. The samples were stained with DAPI and observed at the indicated time points post inoculation. The arrows highlight the accelerated appressorial development in the *pdeH*Δ, while the asterisk (*) indicates a nucleus in mitosis. Scale Bar = 10 micron.
(3.21 MB TIF)

Figure S5 Membrane localization of RFP-PdeH and the perinuclear distribution of PRO*_Mpg1_*-GFP-PdeH. (A) Conidia harvested from the RFP-PdeH expressing strain was treated with tricyclazole (melanin biosynthesis inhibitor) at 0 h and incubated on inductive plastic cover slips for 6 h prior to microscopic observations. The arrows indicate the probable plasma membrane localization of RFP-PdeH (including cytosolic foci). (B) Conidia from the strain expressing PRO*_Mpg1_*-GFP-PdeH were harvested and inoculated on plastic cover slips in a moist chamber. The samples were stained with Hoechst 33342 (nuclei) and visualized by epifluorescence microscopy. PRO*_Mpg1_*-GFP-PdeH is excluded from the nucleus but is predominantly present as perinuclear, cytoplasmic punctae or foci (arrows). Scale Bar = 10 micron.
(4.35 MB TIF)

Video S1 GFP-PdeH in germinating conidia (2–3 hpi)
(1.05 MB MOV)

Video S2 GFP-PdeH in germ tubes at the hooking stage (4–5 hpi)
(0.86 MB MOV)

Video S3 GFP-PdeH in a developing appressorium (6–7 hpi)
(0.32 MB MOV)
