# Supplementary figures and images for: PdeH, a High-Affinity cAMP Phosphodiesterase, Is a Key Regulator of Asexual and Pathogenic Differentiation in Magnaporthe oryzae
Source: PLoS Pathog. 2010 May 6;6(5):e1000897. doi: 10.1371/journal.ppat.1000897 (PMC2865543; doi:10.1371/journal.ppat.1000897)

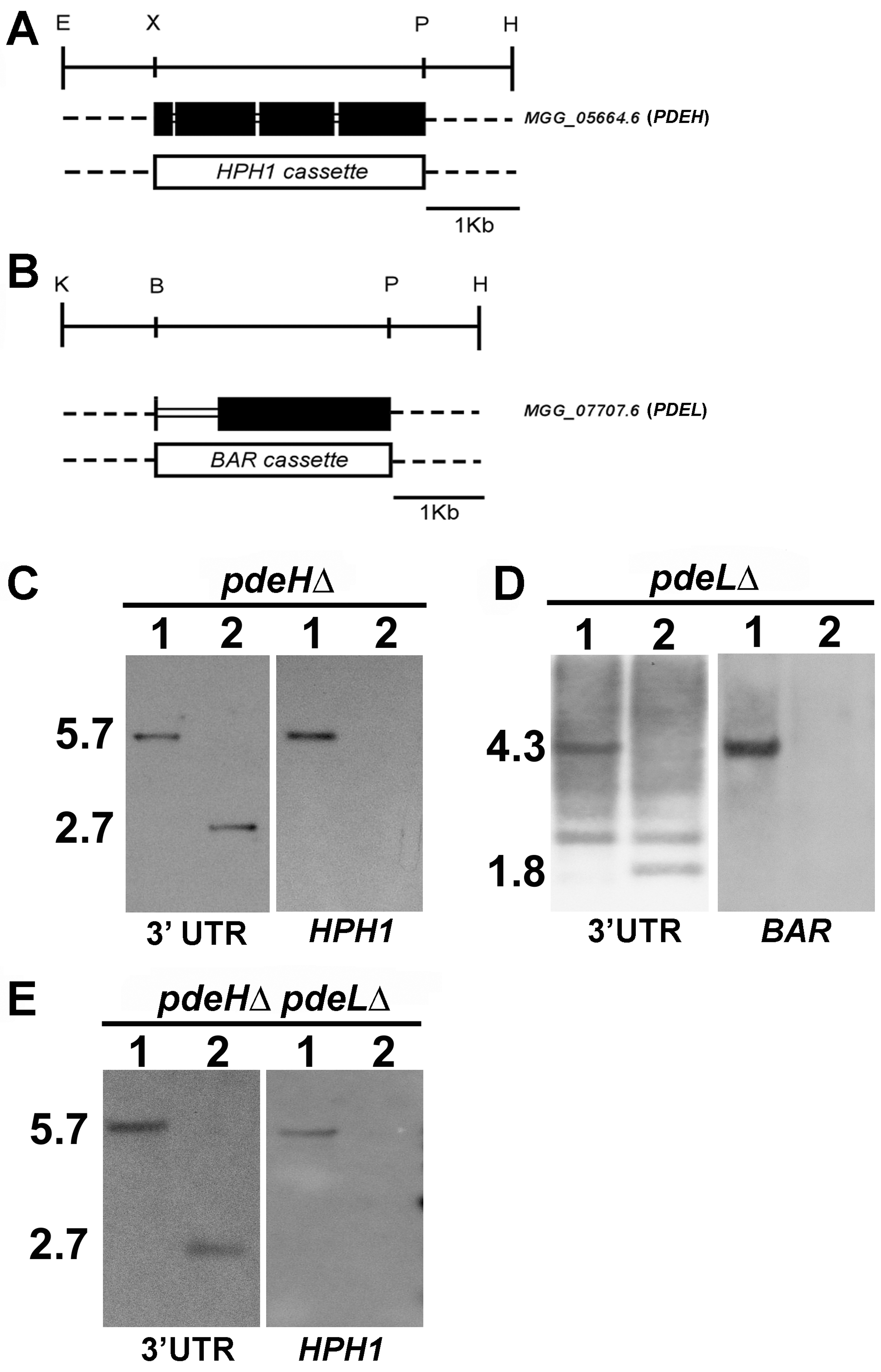

Supplement: Figure S1 — Schematic representation and deletion analysis of PDE genes in Magnaporthe oryzae. (A) Diagrammatic presentation of the ScPDE2 ortholog MGG_05664 in M. oryzae. Solid bars and short open boxes represent coding regions and introns (respectively), while dashed lines indicate the genomic flanks used for gene targeting. Restriction enzyme sites used for cloning are depicted (E: EcoRI, X: XbaI, P: PstI and H: HindIII) and HPH1 refers to the hygromycin-resistance gene cassette used for gene replacement. Scale bar equals 1 kb and delineates the probe used for Southern blot analysis. (B) Diagrammatic representation of the ScPDE1 ortholog MGG_07707 in M. oryzae. The solid bars indicate exons while open boxes depict introns. Dashed lines indicate the flanks used for targeted gene disruption. K: KpnI, B: BamHI, P: PstI and H: HindIII are the restriction enzyme sites used for cloning, and BAR refers to the bialaphos-resistance gene cassette. Scale bar represents 1 kb and the probe used for Southern analysis. (C) Southern analysis for confirmation of the PDEH deletion strains. Genomic DNA from the wild type or pdeHΔ was digested with XhoI and probed with a 1 kb fragment representing the 3′UTR region. The appearance of a 5.7 kb fragment in the deletion strain (lane 1) and a 2.7 kb in the control wild type (lane 2), indicated an accurate PDEH replacement event. The blot was stripped and was re-probed with HPH1 to detect the diagnostic 5.7 kb band in the pdeHΔ. (D) DNA gel blot analysis to confirm the PDEL deletion. NcoI-digested genomic DNA extracted from wild type or pdeLΔ was probed with the PDEL 3′UTR fragment. Presence of the 4.3 kb fragment in the mutant (lane 1) and a 1.8 kb fragment in the wild type (lane 2), indicates precise PDEL replacement. The membrane was re-probed with BAR to further confirm the diagnostic 4.3 kb fragment in the pdeLΔ. (E) Confirmation of the pdeHΔ pdeLΔ strain. The double deletion mutant was created by deleting the PDEH gene in the pdeLΔ background. [file ppat.1000897.s001.tif]

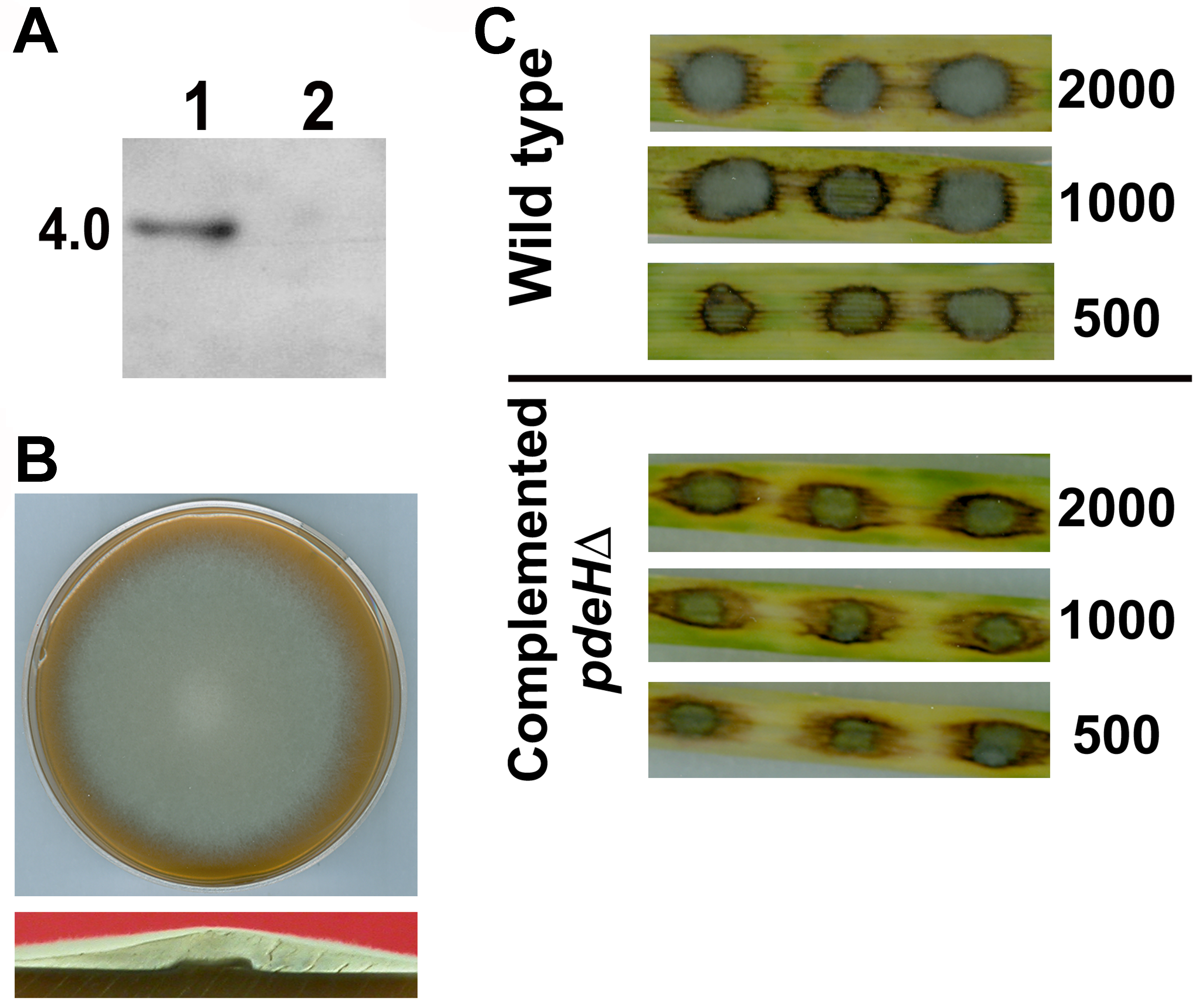

Supplement: Figure S2 — Genetic complementation analysis of the pdeHΔ strain. (A) Southern blot analysis of the RFP-PdeH expressing pdeHΔ strain. The RFP-PdeH expressing pdeHΔ strain was generated by transforming an in-frame RFP-PDEH translational fusion construct to integrate in the pdeHΔ background. NcoI digested genomic DNA from the RFP-PdeH expressing pdeHΔ (lane 1) or the wild type (lane 2) was subjected to Southern blotting with an RFP specific probe. Copy number of the integron (complementation cassette) was judged by southern analysis. The size of the relevant fragment in kilo base-pair is depicted. (B) Growth and colony characteristics of the RFP-PdeH expressing pdeHΔ strain (upper panel) grown for a week in the dark on prune agar medium. The lower panel shows a medial cross section of the above colony depicting significant restoration of aerial hyphal growth. (C) The RFP-PdeH expressing pdeHΔ regained the ability to cause blast disease. Barley leaf explants were inoculated with indicated number of conidia (in triplicate) from the wild type or the RFP-PdeH expressing pdeHΔ. The lesions formed were scored 7 d post inoculation. The RFP-PdeH expressing pdeHΔ formed typical disease lesions comparable to the wild type at the respective conidial dilutions tested. (6.58 MB TIF) [file ppat.1000897.s002.tif]

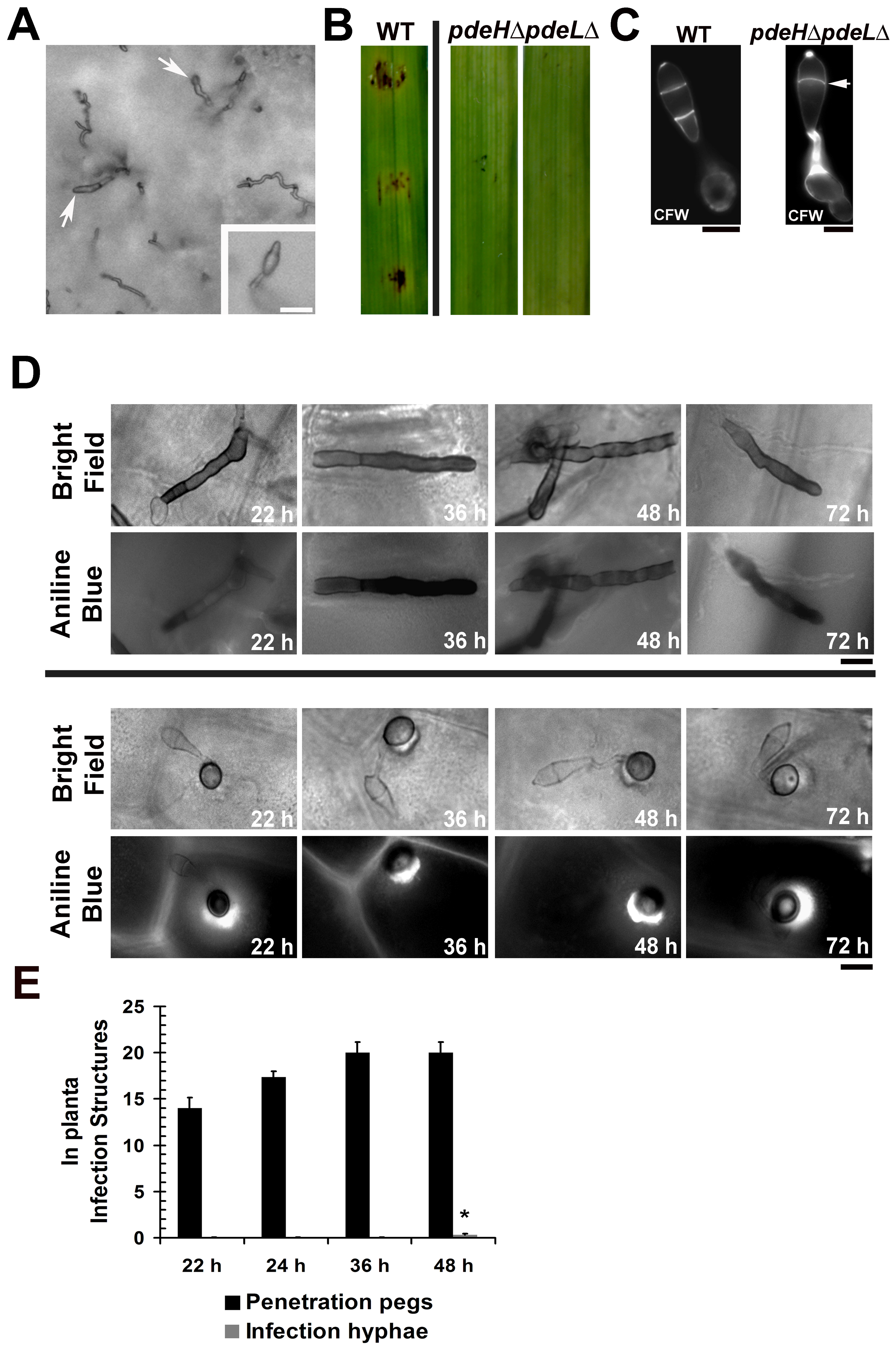

Supplement: Figure S3 — Characterization of the pdeHΔ pdeLΔ mutant. (A) Conidiation is severely reduced upon loss of PDE genes. Close up view of the surface of the pdeHΔ pdeLΔ colony showing aberrant conidiation-related aerial structures (arrows). Inset depicts the rare conidia-like structure formed by the pdeHΔ pdeLΔ mutant. Scale bar = 10 micron. (B) Barley leaf explants inoculated with aberrant conidiation structures from pdeHΔ pdeLΔ or conidia from the wild type (WT) were analyzed 7 dpi. (C) Conidia from the wild type or double deletion mutant were inoculated on coverslips for 24 h and stained with calcofluor white prior to epifluorescence imaging. The arrow highlights the single septa in the conidia formed by the double deletion mutant. Scale bar = 10 micron. (D) The aberrant structures formed during the conidiation phase in the pdeHΔ pdeLΔ do not elicit a host response, unlike the two celled conidia. Photomicrographs depicting aniline-blue stained host papillary callose deposits in the pdeHΔ pdeLΔ strain at the indicated time points post inoculation. Scale bar = 10 micron. (E) Bar chart representing effective host penetration (black bars) as well as the elaboration of infection hyphae (gray bars) by the two celled conidia (n = 25) in the double deletion mutant. However the resultant penetration fails to elaborate/develop infection hyphae in the host. Asterisk highlights the solitary infection hypha detected at 48 h. Values represent mean ± S.E from two independent experiments involving 25 conidia per sample. (8.01 MB TIF) [file ppat.1000897.s003.tif]

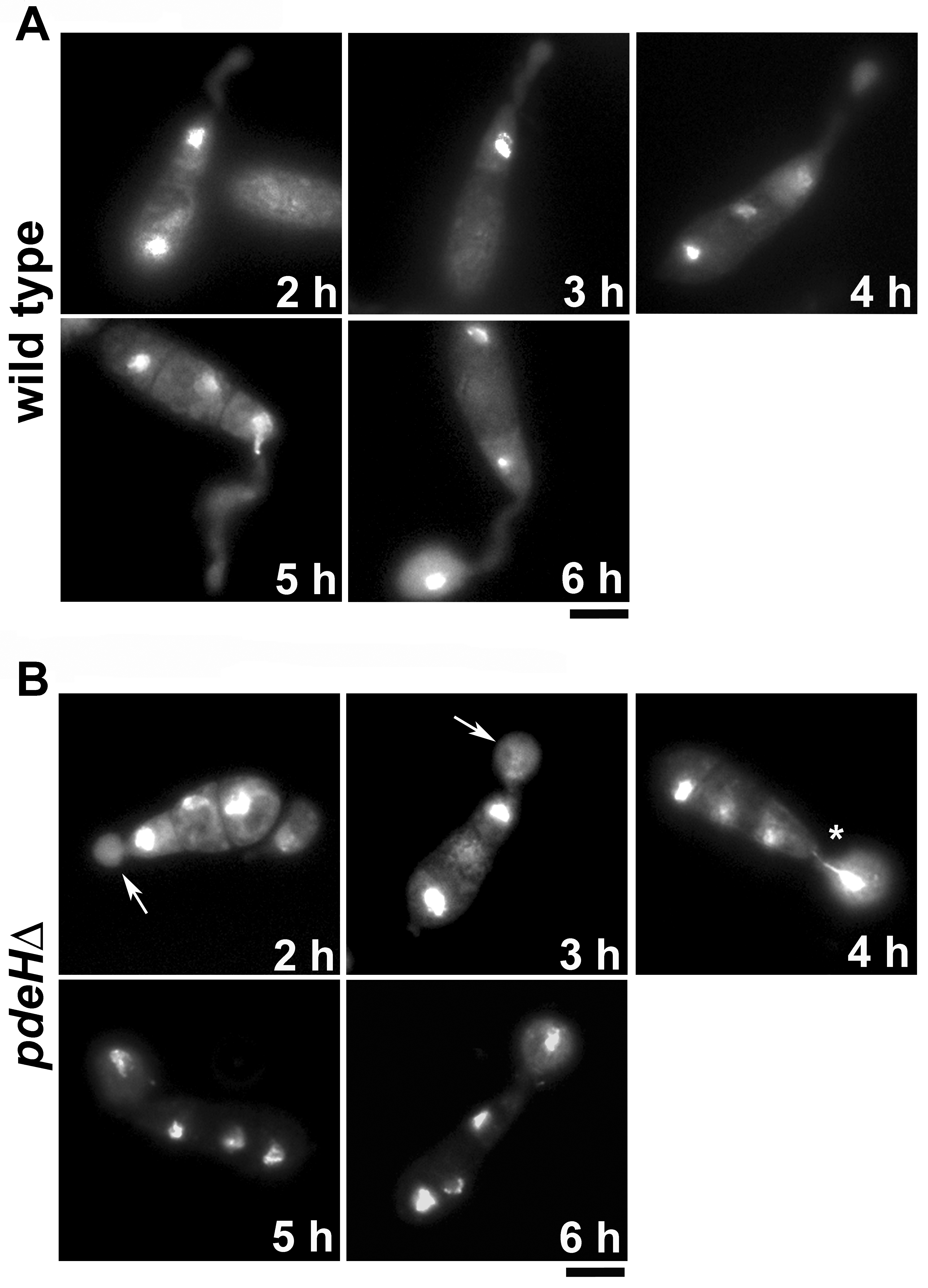

Supplement: Figure S4 — Analysis of nuclear division during appressorial morphogenesis in M. oryzae. (A) and (B) conidia from the wild or the pdeHΔ were inoculated on plastic cover slips in a moist chamber. The samples were stained with DAPI and observed at the indicated time points post inoculation. The arrows highlight the accelerated appressorial development in the pdeHΔ, while the asterisk (*) indicates a nucleus in mitosis. Scale Bar = 10 micron. (3.21 MB TIF) [file ppat.1000897.s004.tif]

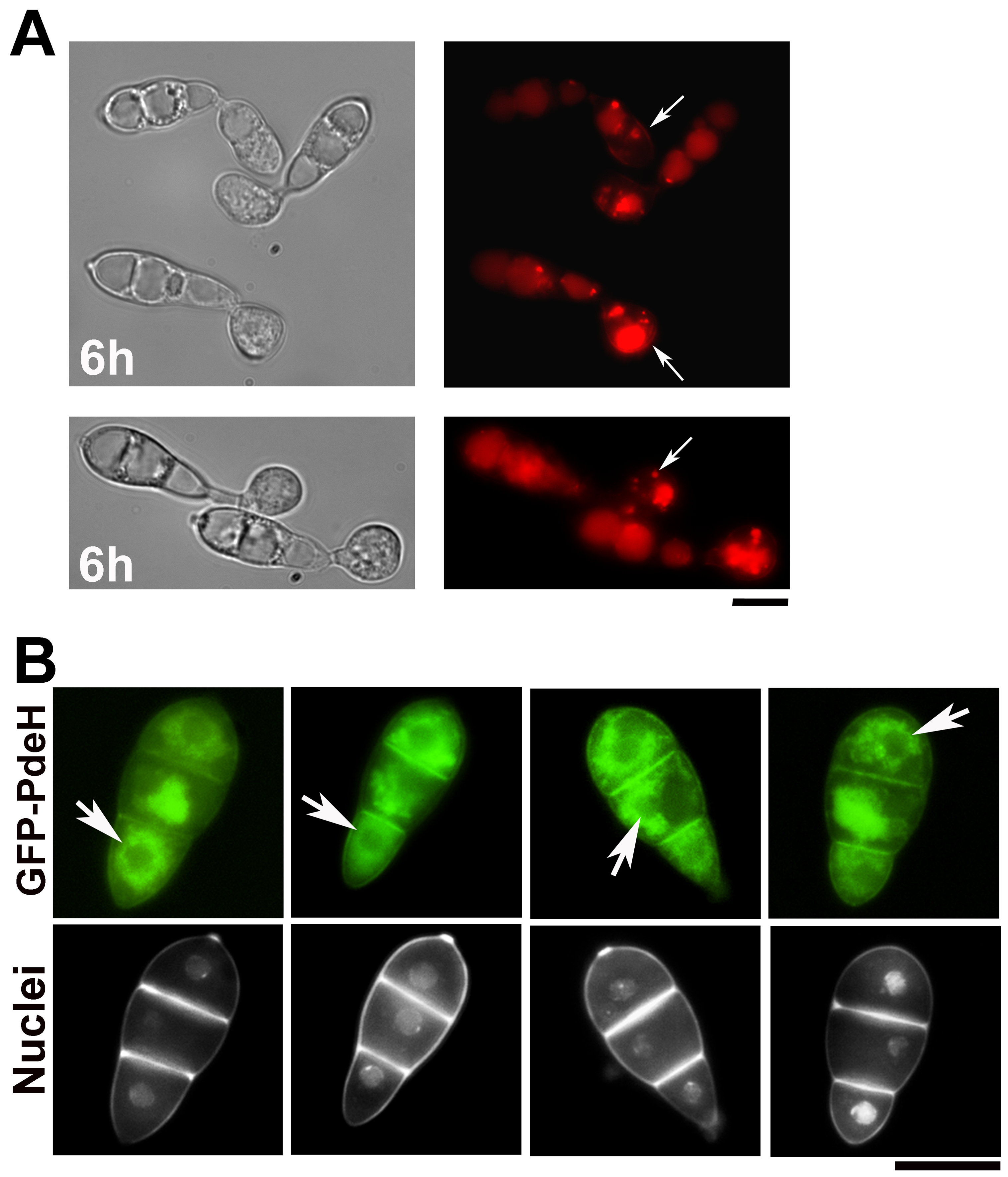

Supplement: Figure S5 — Membrane localization of RFP-PdeH and the perinuclear distribution of PROMpg1-GFP-PdeH. (A) Conidia harvested from the RFP-PdeH expressing strain was treated with tricyclazole (melanin biosynthesis inhibitor) at 0 h and incubated on inductive plastic cover slips for 6 h prior to microscopic observations. The arrows indicate the probable plasma membrane localization of RFP-PdeH (including cytosolic foci). (B) Conidia from the strain expressing PROMpg1-GFP-PdeH were harvested and inoculated on plastic cover slips in a moist chamber. The samples were stained with Hoechst 33342 (nuclei) and visualized by epifluorescence microscopy. PROMpg1-GFP-PdeH is excluded from the nucleus but is predominantly present as perinuclear, cytoplasmic punctae or foci (arrows). Scale Bar = 10 micron. (4.35 MB TIF) [file ppat.1000897.s005.tif]
